# Atypical chemokine receptor ACKR3/CXCR7 controls postnatal vasculogenesis and arterial specification by mesenchymal stem cells via Notch signaling

**DOI:** 10.1038/s41419-020-2512-2

**Published:** 2020-05-04

**Authors:** Sung-Tai Wei, Yen‐Chih Huang, Mei-Ling Hsieh, Yu-Jung Lin, Woei-Cherng Shyu, Hui-Chen Chen, Chia-Hung Hsieh

**Affiliations:** 1 Graduate Institute of Biomedical Sciences, China Medical University, Taichung, Taiwan; 2 Department of Neurosurgery, China Medical University and Hospital, Taichung, Taiwan; 3 Department of Medical Imaging, China Medical University and Hospital, Taichung, Taiwan; 4 Graduate Institute of Immunology, China Medical University, Taichung, Taiwan; 5 Department of Medical Research, China Medical University Hospital, Taichung, Taiwan; 6 Department of Biomedical Informatics, Asia University, Taichung, Taiwan

**Keywords:** Morphogen signalling, Mesenchymal stem cells

## Abstract

Mesenchymal stem cells (MSCs) are known to play a role in postnatal vasculogenesis and hold great promise for vascular regeneration. However, the mechanisms by which the endothelial differentiation and specification of MSCs remain unclear. We examined the potential role and molecular mechanisms of atypical chemokine receptor ACKR3/CXCR7 in MSC-mediated endothelial cell differentiation and specification. Here, we showed that vascular endothelial growth factor (VEGF) and platelet-derived growth factor (PDGF) activate CXCR7 expression on MSCs through PDGF receptors, PDGFRα and PDGFRβ-mediated phosphoinositide 3-kinase (PI3K)/Akt signaling. Genetic and pharmacologic blockage of CXCR7 on MSCs suppressed the VEGF or stromal cell-derived factor 1 (SDF)-1-induced the capacity for vasculogenesis in vitro and in vivo. Moreover, CXCR7 gain of function markedly promoted vasculogenesis by MSCs in vitro and in vivo and induced endothelial differentiation along the arterial endothelial cell lineage via upregulation of Notch signaling. However, blockade of Notch signaling inhibited CXCR7-induced vasculogensis by MSCs. These results indicate CXCR7 is a critical regulator of MSC-mediated postnatal vasculogenesis and arterial specification via Notch signaling.

## Introduction

Mesenchymal stem cells (MSCs) are a population of self-renewing and multipotent cells capable of differentiating into multiple cell types, including osteocytes, chondrocytes, adipocytes, hepatocytes, myocytes, neurons, cardiomyocytes, and endothelial cells^1,2^. These cells were originally isolated from the bone marrow stroma, but they have recently been identified also in other tissues, such as fat, epidermis, and cord blood. Accumulating evidence reveals that MSCs play an important role in new blood vessels in the adult, a process known as postnatal vasculogenesis, especially during tissue ischemia and tumor vascularization^3–5^. Therefore, exploring mechanisms which regulate the role of MSCs in vasculogenesis is a key therapeutic objective for increasing neovascularization in tissue ischemia or inhibiting vessel formation in tumors.

Vascular endothelial growth factor (VEGF) and platelet-derived growth factor (PDGF) play crucial roles in regulating angiogenesis^6^, but their involvement in regulating MSCs during vasculogenesis is less well understood. Although the mechanism is not completely understood, it has been reported that VEGF can induce MSCs differentiated into endothelial cells (ECs)^7,8^. Besides, knockout studies have demonstrated crucial roles for the PDGF-B and its cognate receptor, platelet-derived growth factor receptor-β (PDGFR-β), in blood vessel maturation^9^. PDGF receptors could also stimulate the sprouting vasculogenesis in differentiating embryonic stem cells^10^, suggesting PDGF/PDGFR signaling is involved in vasculogenesis during embryonic development. Despite the fact that MSCs did not express VEGF receptors, VEGF-A can stimulate both PDGFRα and PDGFRβ tyrosine phosphorylation activation and further promote MSC migration and proliferation^11^. VEGF-A/PDGF receptor signaling mechanism may also in directing MSCs toward a vascular cell fate^3^.

Atypical chemokine receptor CXCR7 (ACKR3) functions as a scavenger receptor for chemokine CXCL12^12,13^ or trigger β-arrestin-dependent signaling^14^, a molecule that promotes developmental and pathological angiogenesis^15^. Although the role of CXCR7 in postnatal vasculogenesis is unknown, CXCR7 knockout mice exhibit postnatal lethality within a week after birth owing to severe cardiovascular defects, supporting the notion that CXCR7 plays a key role in the cardiovascular system at multiple points in development^16^. Morpholino-mediated knockdown of CXCR7 in zebrafish also supports a critical role for CXCR7 in developmental vascular formation and angiogenesis^17^. However, CXCR7 levels in normal healthy human hematopoietic stem cells or other stem cells are very low^18,19^. Therefore, the link between CXCR7 and endothelial differentiation of stem cells remains obscure and controversial.

Here, we show that VEGF or PDGF induces CXCR7 expression on MSCs through PDGF receptors, PDGFRα and PDGFRβ-mediated phosphoinositide 3-kinase (PI3K)/Akt, signaling mechanism. Genetic or pharmacological manipulation of CXCR7 in MSCs reveals that CXCR7 is critical regular for MSC-mediated vasculogenesis in vitro and in vivo. Moreover, CXCR7 gain-of-function also markedly promoted arterial endothelial differentiation and vasculogenesis by MSCs in vitro and in vivo via Notch signing. Collectively, our data provide the novel insight into CXCR7 in postnatal vasculogenesis and arterial specification.

## Materials and methods

### Cell cultures

Immortalized clonal cell lines of human MSCs (ihMSCs) and primary bone marrow-derived human MSCs (phMSCs) were derived as described previously^20,21^. MSCs were cultured in the basic medium (α-MEM, Gibco) supplemented with 10% fetal bovine serum (FBS, Gibco), 100 U/ml penicillin (GIBCO), 10 μg/ml streptomycin (GIBCO), 2 mM L-glutamines (GIBCO), 0.2 mM L-ascorbic acid 2-phosphate magnesium salt (ASAp, Sigma-Aldrich) at 37 °C in a humid atmosphere with 5% CO_2_. Clonetics™ Human Skeletal Muscle Cells (SkMC) were purchased from Lonza and maintained in Clonetics SkGM Bullet kit (Lonza).

### Growth factors and inhibitors

Human recombinant human VEGF_165_ and PDGF-BB were purchased from R&D Systems. SDF-1 was purchased from UPSTATE. Neutralizing anti-human and mouse VEGF antibodies were purchased from R&D Systems. Neutralizing anti-human PDGFRα and PDGFRβ antibodies were purchased from R&D Systems. U-73122, U0126, LY294002, AMD3100, and DBZ were purchased from Sigma-Aldrich. CCX771 was a kind gift from Dr. Mark Penfold (ChemoCentryx).

### Mouse GFP+MSCs isolation

Mouse GFP^+^MSCs were isolated and cultured according to the protocol^22^. Bone marrow cell were collected from GFP transgenic mice by flushing the femurs and tibias with RPMI medium supplemented with 10% heat-inactivated FBS, 50 IU/ml penicillin and 50 µg/ml streptomycin (Invitrogen). Erythrocytes were depleted by hypotonic lysis. Bone marrow cells were plated at a density of 1 × 10^6^ cells per cm^2^ in RPMI complete medium. Culture medium was changed and washed by 1× PBS every day to remove non-adherent cells. GFP^+^MSCs were grown until confluent. Adherent cells were then detached by 0.25% trypsin-EDTA and replated using a 1∶4 dilution. Subsequent passaging was performed at a density of 10,000 cells per cm^2^. GFP^+^MSCs were used after three passages.

### Identification and characterization of GFP^+^MSCs

Mouse GFP^+^MSCs were incubated with 1 µg of phycoerythrin (PE)-conjugated antibodies (CD29, CD73, CD105, and CD34; BD Bioscience) or isotype-matched negative control antibody at 4 °C for 45 min according to the manufacturer’s instructions. Samples were analyzed by a fluorescence-activated cell sorting (FACS) Calibur flow cytometer (BD Bioscience). GFP^+^MSCs were induced osteogenesis, chondrogenesis, and adipogenesis by using STEMPRO^®^ osteogenesis, chondrogenesis, and adipogenesis differentiation kits (GIBCO). Medium was replaced every 4 to 7 days. Differentiation was terminated after 28 days. After the appearance of morphologic features of differentiation, cells were stained with Alizarin Red, Alcian Blue, and Oil Red (Sigma-Aldrich) for osteocytes, chondrocytes, and adiopocytes, respectively.

### Isolation of implanted GFP^+^MSCs

Tissues were disaggregated with an enzyme cocktail containing collagenase type III (Sigma-Aldrich), hyaluronidase (Sigma-Aldrich), and collagenase type IV (Sigma-Aldrich), washed several times, and resuspended in phosphate-buffered saline (PBS) to produce a single-cell suspension. GFP was measured using a FACScalibur instrument and FACSDiva 6.0 software (BD Bioscience). GFP^+^ cells were gated and isolated according to GFP expression and side scatter (SSC).

### In vitro hypoxic treatment

Cells were treated in a Biospherix C-Chamber (Biospherix) inside a standard culture chamber by means of exhausting and gassing with 95% N_2_ and 5% CO_2_ to produce oxygen concentrations of 0.5 to 1% at 37 °C to achieve hypoxic condition. Cells incubated in hypoxia condition for 24 h.

### Enzyme-linked immunosorbent assay (ELISA)

Antibody sandwich ELISAs were used to evaluate VEGF levels in the conditional medium (CM) according to the manufacturer’s instructions (Sigma-Aldrich).

### Western blot analysis

Cell extracts were prepared as described previously^23^. In total, 30 µg of proteins were loaded and electrophoresed using SDS-PAGE gels then transferred to a PVDF membrane. The membrane was blocked for 1 h using blocking solution, then was incubated with the primary antibody overnight at 4 °C. The following antibodies were used: β-actin (A5316, Sigma-Aldrich, 1:10,000 dilution), CXCR7 (GTX100027, GeneTex Inc., 1:500 dilution), PDGFRα (MAB322, R&D Systems, 1:2000 dilution), PDGFRβ (MAB1263, R&D Systems, 1:1000 dilution), VEGFR1 (AF321, R&D Systems, 1:1000 dilution), VEGFR2 (AF357, R&D Systems, 1:1000 dilution), VEGFR3 (AF349, R&D Systems, 1:1000 dilution), PLC-γ1 (#2822, Cell Signaling, 1:1000 dilution), phospho-PLC-γ1 (#2821, Cell Signaling, 1:1500 dilution), MEK-1/2 (#9122, Cell Signaling, 1:1000 dilution), phospho-MEK-1/2 (#9121, Cell Signaling, 1:1500 dilution), Akt (#9272, Cell Signaling, 1:1000 dilution), phospho-Akt (#9271, Cell Signaling, 1:1500 dilution), NOTCH1 (ab52627, Abcam, 1:1000 dilution), JAG1 (ab7771, Abcam, 1:1000 dilution), JAG2 (ab226814, Abcam, 1:1000 dilution), DDL4 (MAB1389, R&D Systems, 1:1000 dilution), HEY1 (GTX118007, GeneTex Inc., 1:1000 dilution), EPHB2 (AF467, R&D Systems, 1:1000 dilution) and NRP1 (AF3870,R&D Systems, 1:500). The second day, after three washing steps with TBS-0.05% Tween-20, the blot was incubated with secondary horseradish peroxidase-conjugated antibody (A9917, Sigma-Aldrich, 1:10000 dilution) for 45 min. The blot was washed three times with TBS-0.05% Tween-20, then a super signal west pico chemiluminutesescent substrate (Thermo Scientific) was used for the detection of protein bands. Relative band densities of the various proteins were measured from scanned films using ImageJ Software (NIH).

### Quantitative real-time polymerase chain reaction (Q-PCR)

The total RNA from the cells was obtained using the RNeasy Minutesi Kit (Qiagen) according to the manufacturer’s protocol, and reverse-transcribed with Omniscript RT (Qiagen) using random hexamers (Applied Biosystems). Quantitative PCR was performed in an Opticon 2 Monitor (MJ Research) and SYBR Green I dye (Applied Biosystems). For Q-PCR primer sequences, see Supplementary Table 1. The average of each gene cycle threshold (Ct) was determined for each experiment. Relative cDNA levels (2^−ΔΔCt^) for the genes of interest were determined using the comparative Ct method, which generates the ΔΔCt as the difference between the gene of interest and the housekeeping gene 18S rRNA for each sample. Each averaged experimental gene expression sample was compared with the averaged control sample, which was set to 1.

### Flow cytometric analysis

Surface expression of CXCR7, Flk-1, Flt-1, vWF, VE-cadherin, and CD31 was evaluated by flow cytometric analysis. Cells were harvested with PBS containing 5 mM EDTA and immediately neutralized in FACS buffer (α-MEM containing 1% BSA and 0.025% NaN_3_). After extensive washing with FACS buffer, cells (10^5^ cells) were incubated with 1 µg/ml of the primary antibodies, including CXCR7 (MAB42273, R&D Systems, 1:100 dilution), Flk-1 (Avas12a1, Novus, 1:100 dilution), Flt-1 (MAB321, R&D System, 1:100 dilution), vWF (ab8822, Abcam, 1:100 dilution), VE-cadherin (FAB9381P, R&D System, 1:100 dilution), and CD31 (FAB806G, R&D Systems, 1:100 dilution) by shaking for 1 h at 4 °C. After extensive washing with FACS buffer, cells were incubated with DyLight 649 AffiniPure goat anti-rabbit IgG or DyLight 488 AffiniPure goat anti-mouse IgG (115-495-209 or 111-545-144, Jackson Immunoresearch, 1:100 dilutions) by shaking for 1 h at 4 °C. Cells were then washed with FACS buffer five to six times, and fixed in PBS containing 1% paraformaldehyde. Expression levels were measured on a FACScalibur instrument and FACSDiva 6.0 software (BD Bioscience).

### Plasmids and shRNA

To construct pAS2.CXCR7-Puro-H and pAS2.CXCR7-Puro-M, human and mouse total RNAs were extracted from MCF7 cells and mouse epithelial cells using RNeasy kit (Qiagen), respectively. In total, 500 ng of total RNA was used in reverse transcription reaction, and cDNA was generated with Superscript II reverse transcriptase (Invitrogen). Full-length human CXCR7 cDNA and mouse CXCR7 cDNA were amplified in a reaction with Platinum Taq DNA polymerase (Invitrogen) using the human CXCR7 primers and mouse CXCR7 primers, as described previously^24,25^, which harboring 5′ NheI and 3′ and EcoRI sites. The fragments were subcloned into pAS2.EYFP.puro (National RNAi core facility, Academia Sinica, Taiwan) at the NheI and EcoRI sites, respectively, and then the cDNA sequences were confirmed. Lentiviral vectors carrying short hairpin RNAs (shRNAs)-targeting CXCR7 and scrambled shRNA (http://rnai.genmed.sinica.edu.tw/file/vector/C6-7/17.1.pLAS.Void.pdf) were provided by National RNAi core facility, Academia Sinica in Taiwan. The detailed shRNA target sequences used in this study are described in Supplementary Table 2. The pGreenFire1-SFFV^26^ was used to generate MSC reporter cells bearing SFFV promoter-driven dual optical reporter gene encoding both green fluorescence protein (GFP) and luciferase (Luc).

### Lentivirus production and transduction

Lentiviral particles were generated by transiently cotransfecting 293T cells with the plasmids coding for CXCR7 (pAS2.CXCR7-Puro-H; pAS2.CXCR7-Puro-M), GFP (pLAS2.1w.PeGFP-I2-Bsd), scrambled shRNA and shRNAs-targeting PDGFRα, PDGFRβ, and CXCR7 in addition to plasmids encoding gag/pol and VSV-G envelope genes. Transfection was carried out with jetPEI reagent (Polyplus-Transfection). Subconfluent cells were infected with lentivirus in the presence of 8 μg/ml polybrene (Sigma-Aldrich). At 24 h post infection, medium was removed and replaced with fresh growth medium containing puromycin (0.5 µg/ml) select for infected cells after 48 h post infection.

### siRNA transfection

MSCs with siRNA transfections were carried out using Lipofectamine 2000 reagent (Invitrogen) according to the manufacturer’s instructions. MSCs were transfected with either PDGFRα siRNA, PDGFRβ siRNA, or a scrambled siRNA as a control (QIAGEN).

### VEGF or SDF-1-induced differentiation

MSCs were cultivated in the presence of 2% FCS and 12 ng/ml recombinant human VEGF_165_ (R&D Systems) or 100 ng/ml SDF-1 (UPSTATE) for 7 days. Medium was changed every 2 days. For inhibitors studies, the medium was additionally supplemented with AMD3100 (10 μM; Sigma-Aldrich) and CCX771 (100 nM; ChemoCentryx).

### In vitro tube-formation assay

Wild-type MSCs, scramble shRNA-expressing MSCs or CXCR7 shRNA-expressing MSCs were treated with VEGF (12 ng/ml) medium for 7 days. Wild-type MSC, control vector-expressing MSCs, or CXCR7-expressing MSCs were cultivated in normal medium for 7 days. After 7 days, Matrigel (growth factor reduced; BD Biosciences) was spread on a 96-well polystyrene plate and allowed to solidify at 37 °C. Cells were plated on Matrigel-covered plates in VEGF (12 ng/ml) medium or normal medium. After 16 h, the tube structure of the plated cells was observed using a Zeiss observer A1 axio microscope (Zeiss). For quantitative measurements of capillary tube formation, Matrigel wells were digitized under a ×4 objective for measurement of total tubes and tube length of capillary tube formation. Tracks of endothelial cells organized into networks of cellular cords (tubes) were counted and averaged in randomly selected five microscopic fields.

### Luciferase reporter assay

To examine the SDF-1/CXCR7 axis-mediated CBF1 reporter activities, a traditional dual-luciferase assay consisting of four Notch-sensing CBF1-binding sites reporter (normalized to a control promoter driving Renilla luciferase) was used, as previously described^27^. pCBFRE-luc was a gift from Nicholas Gaiano (Addgene plasmid # 26897). Briefly, cells were cotransfected with CBF1-luciferase reporter construct and Renilla reporter plasmid. At 24 h after transfection, the luciferase activity was examined by a dual-luciferase reporter assay system (Promega) according to the manufacturer’s instructions, and firefly luciferase activity was normalized to the control renilla activity included in the kit. Luciferase activities are expressed as fold increase over the luciferase activities in un-stimulated conditions.

### In vivo Matrigel plug assay

Mice were anesthetized in a 3% isoflurane-saturated chamber, and maintained by a facemask. To examine the effect of CXCR7 gain-of-function on MSC-mediated vasculogenesis in vivo, 2 × 10^6^ wild-type GFP^+^MSCs, control vector-expressing GFP^+^MSCs or CXCR7-expressing GFP^+^MSCs were resuspended in 500 μl Matrigel (BD Bioscience) and implanted subcutaneously in C57BL/6 mice (male, 6–8 weeks of age). On the other hand, to observe the role of CXCR7 on MSCs in VEGF-mediated vasculogenesis, 2 × 10^6^ wild-type GFP^+^MSCs, scramble shRNA-expressing GFP^+^MSCs, or CXCR7 shRNA-expressing GFP^+^MSCs were mixed with 500 μl Matrigel (BD Bioscience) containing 50 ng/ml VEGF and implanted subcutaneously in C57BL/6 mice. After 2 weeks, the Matrigel plugs were removed for hemoglobin content and immunofluorescence imaging assays.

### Hemoglobin content assay

Amount of hemoglobin (Hb) was measured using Hemoglobin Colorimetric Assay Kit (Cayman Chemical) according to the manufacturer’s instructions.

### Immunofluorescence and histochemistry

Matrigel plugs or ischemic limbs were frozen in the OCT embedding matrix (Shandon Lipshaw). Frozen tissue sections (10 μm) were obtained with an OTF cryomicrotome (Bright-Hacker), fixed in ice-cold methanol for 10 min, and washed with PBS. Tissue sections were stained for 2 h with the following antibodies: CD31 (BD Pharmingen), vWF (Millipore), or EPHB2 (R&D Systems). DyLight 649-conjuated antibodies were used for secondary detection. After washing in PBS, nuclear labeling was obtained with DAPI (Invitrogen). Cover slides were mounted and analyzed using an Axio observer A1 digital fluorescence microscope system (ZEISS). Six mice from each group were analyzed. Five sections were randomly collected from each mouse, and calculated the average positive immunoactivity. For histochemistry, tissues were paraformaldehyde fixed, paraffin embedded, and sectioned for standard hematoxylin & eosin (H&E) staining, as described previously^28^.

### Hind-limb ischemia and cell transplantation

All animal studies were conducted according to the Institutional Guidelines of China Medical University and approved by the Institutional Animal Care and Use Committees of China Medical University (Approved number: 102-92-N). The hind-limb ischemia was induced in C57BL/6 mice (male, 6–8 weeks of age). The femoral artery was excised from its proximal origin as a branch of the external iliac artery to the distal point where it bifurcated into the saphenous and popliteal arteries. After arterial ligation, a total of 5 × 10^6^ GFP^+^MSCs (200 μl) was injected intramuscularly into four sites of the gracilis muscle in the medial thigh. The animals were treated with VEGF n.a. or control IgG at 10 mg/kg i.p. after cell transplantation. Hindlimbs were excised for the isolation of mouse GFP^+^MSCs at 2 days after treatments.

### Statistical analysis

All data are given as mean ± SD. Statistical analyses were performed with SPSS 18.0 software using unpaired Student’s *t* test and ANOVA with Bonferroni’s or Tukey’s multiple comparison post hoc tests, where appropriate.

## Results

### Skeletal muscle cells-secreted VEGF promotes the upregulation of CXCR7 in MSCs

**Fig. 1:**
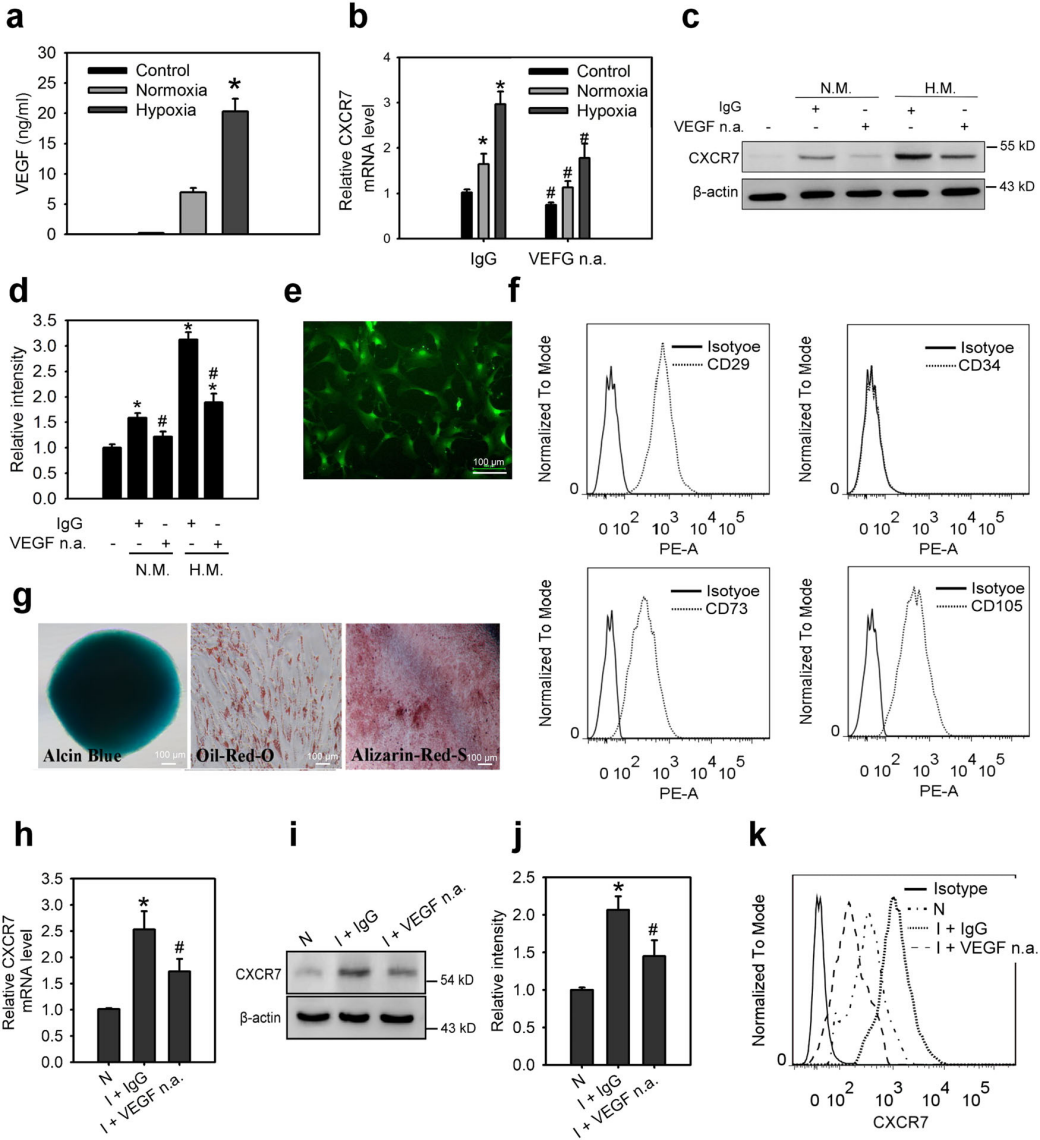
Skeletal muscle cells-secreted VEGF promotes the upregulation of CXCR7 in MSCs. **a** The VEGF concentration in control medium and conditional medium (CM) from SkMC cells incubated in normoxia (N.M.) and hypoxia (H.M.) condition for 24 h. Concentrations of VEGF were examined using ELISA. Data are means ± SD (*n* = 9). *p < 0.01 compared with the control (untreated) group. The mRNA levels (**b**), protein levels (**c**), relative protein densities (**d**) of CXCR7 in ihMSCs incubated with control medium or CM collected from indicated condition that was treated with or without neutralizing anti-VEGF antibody (VEGF n.a., 100 ng/ml) for 18 h. Data are means ± SD (*n* = 9). *p < 0.01 compared with the control (untreated) group. ^#^*p* < 0.01 compared with IgG-treated groups. **e** The morphology characteristics of mouse GFP^+^MSCs. **f** Cell surface co-expression of the antigens CD29, CD34, CD73, and CD105 in mouse GFP^+^MSCs. **g** Differentiation potential of mouse GFP^+^MSCs in osteogenic, chrondrogenic, and adiogenic lineages using Alizarin red, Alcian blue, and Oil red staining, respectively. The mRNA levels (**h**), protein levels (**i**), relative protein densities (**j**), and cell surface expression (**k**) of CXCR7 in mouse GFP^+^MSCs isolated from normal lindlimbs (N) or ischemic hindlimbs (I) with neutralizing anti-VEGF antibody or control IgG treatment via a flow sorting of GFP-expressing cells. Animals were treated with VEGF n.a. or control IgG at 10 mg/kg i.p. Hindlimbs were excised for the isolation of mouse GFP^+^MSCs at 2 days after treatments. Data are means ± SD (*n* = 9). *p < 0.01 compared with the IgG-treated group.

Our first objective was to investigate whether VEGF secreted by human skeletal muscle cells (SkMC) plays a role in the regulation of CXCR7 expression in the immortalized human bone marrow stromal cells (ihMSCs). Hypoxic stress was used to induce the production and secretion of VEGF, as it is a hypoxia-responsive gene^29^. Conditional medium (CM) from hypoxia-treated SkMC cells was collected, and the concentration of VEGF in the medium was determined by enzyme-linked immunosorbent assay (ELISA). Increased levels of VEGF were secreted in the hypoxic condition compared with the normoxic condition (Fig. 1a). Increased CXCR7 mRNA and protein levels were exhibited by ihMSCs cultured in CM from normoxia- or hypoxia-treated SkMC compared with ihMSCs cultured in control medium (Fig. 1b–d). CXCR7 expression in ihMSCs cultured with hypoxic CM was significantly higher than in those cultured with normoxic CM. To determine the role of VEGF in CM-mediated CXCR7 induction, neutralizing antibodies of VEGF were used. Suppression of VEGF inhibited CM-induced CXCR7 expression in ihMSCs (Fig. 1c, d). To extend our studies further in vivo, mouse MSCs of green fluorescent protein (GFP) transgenic mice were isolated from the tibia and femur and kept in culture for several passages. The isolated GFP^+^MSCs highly expressed GFP, CD29, CD73, CD105, and lack of expression of CD34 (Fig. 1e, f). These cells had the potential to differentiate along osteogenic, chondrogenic, and adipogenic lineages (Fig. 1g). GFP^+^MSCs were implanted subcutaneously (s.c.) into the ischemic hindlimbs of mice, and these mice were treated with neutralizing antibodies of VEGF for 2 days. At 2 days after cell transplantation, tissues were digested as single-cell suspension for flow cytometric analysis and cell sorting of GFP^+^ cells. Quantitative real-time polymerase chain reaction (Q-PCR), western blot, flow cytometric analysis, and ELISA revealed that ischemia induced CXCR7 expression in hindlimbs and increased VEGF levels in hindlimbs and plasma (Fig. 1h–k; Supplementary Fig. S1). Moreover, the neutralizing anti-VEGF antibody significantly reduced CXCR7 expression in transplanted GFP^+^MSCs. These findings suggest that VEGF secreted by SkMC cells or ischemic tissues plays a crucial role in regulating CXCR7 expression in MSCs.

### PDGFRα and PDGFRβ are essential for VEGF-induced CXCR7 expression in MSCs

**Fig. 2:**
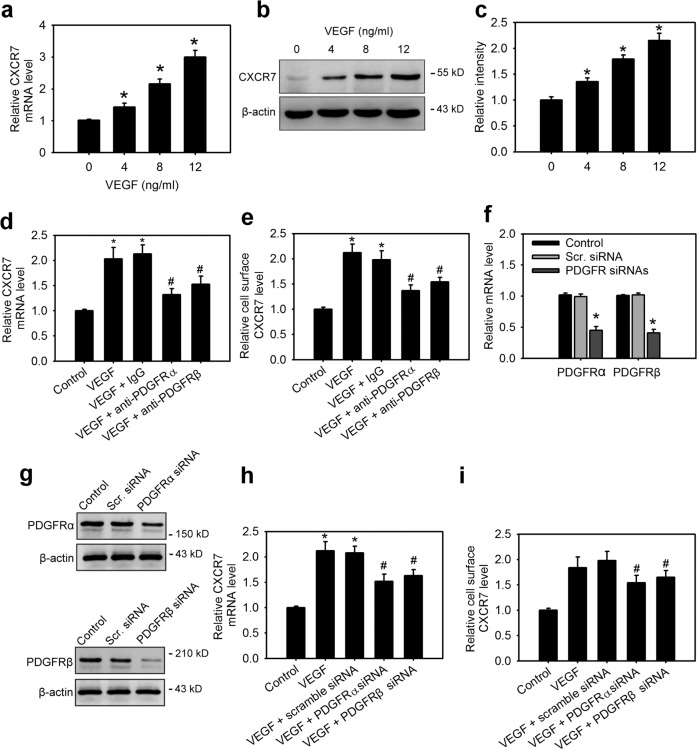
PDGFRα and PDGFRβ are essential for VEGF-induced CXCR7 expression in MSCs. The mRNA levels (**a**), protein levels (**b**), and relative protein densities (**c**) of CXCR7 in ihMSCs incubated with indicated concentrations of VEGF for 18 h. *p < 0.05 compared with the control (untreated) group. The CXCR7 mRNA levels (**d**) and its cell surface levels (**e**) in ihMSCs pretreated with the neutralizing anti-PDGFRα or anti-PDGFRβ antibody followed by stimulation with VEGF (12 ng/ml) for 24 h. Data are means ± SD (n = 9). *p < 0.001 compared with the control (untreated) group. ^#^*p* < 0.01 compared with IgG-treated groups. **f**, **g** Verification of PDGFRα and PDGFRβ knockdown by siRNAs. Data are means ± SD (*n* = 6). *p < 0.001 compared with scramble (Scr.) siRNA. The CXCR7 mRNA levels (**h**) and its cell surface levels (**i**) in ihMSCs transfected with or without scramble (Scr.), PDGFRα or PDGFRβ siRNAs for 24 h followed by stimulation with VEGF (12 ng/ml) for 24 h. Data are means ± SD (*n* = 9). *p < 0.0001 compared to the control (untreated) group. ^#^*p* < 0.01 compared with scramble (Scr.) siRNA.

We next determined which molecular mechanism is involved in VEGF-induced CXCR7 expression in human MSCs. In the ihMSCs cultured with different dosages of human recombinant VEGF, VEGF stimulation elevated CXCR7 expression in a dose-dependent manner (Fig. 2a–c). It has been reported that MSCs did not express VEGF receptors (VEGFRs), but VEGF can signal through platelet-derived growth factor receptors (PDGFRs)^11^. VEGFRs were also not detected in our ihMSCs, but VEGFR1 and VEGFR2 were expressed in human umbilical vein endothelial cells (HUVE) (Supplementary Fig. S2a). Therefore, we next investigated the relationship between PDGFRs and VEGF-induced CXCR7 expression by blocking cell surface PDGFRα or PDGFRβ, using selective neutralization antibodies. ihMSCs were pretreated with either a PDGFRα- or PDGFRβ-specific neutralization antibody before VEGF treatment. Blocking either cell surface PDGFRα or PDGFRβ significantly inhibited VEGF-induced CXCR7 expression (Fig. 2d, e; Supplementary Fig. S2b), with PDGFRα neutralization resulting in greater inhibition of VEGF-mediated CXCR7 expression. To further demonstrate that both PDGFRα and PDGFRβ are crucial receptors in directing VEGF-induced CXCR7 expression, the specific validated siRNA PDGFRα and PDGFRβ nucleotides were used to knockdown the respective transcripts. Q-PCR and western blot analysis confirmed successful knockdown as manifested by significantly decreased PDGFRs expression (Fig. 2f, g; Supplementary Fig. S2c). ihMSCs transfected with scrambled siRNA had no effect on VEGF-induced CXCR7 expression (Fig. 2h, i). However, VEGF-induced CXCR7 expression was effectively attenuated when any PDGFRs including DGFRα and PDGFRβ were silenced (Fig. 2h, i), indicating functional cell surface PDGFRα and PDGFRβ are both crucial determinants in mediating VEGF-induced CXCR7 expression in MSCs.

### PDGFR-mediated PI3K signaling is required for VEGF or PDGF-induced CXCR7 expression

**Fig. 3:**
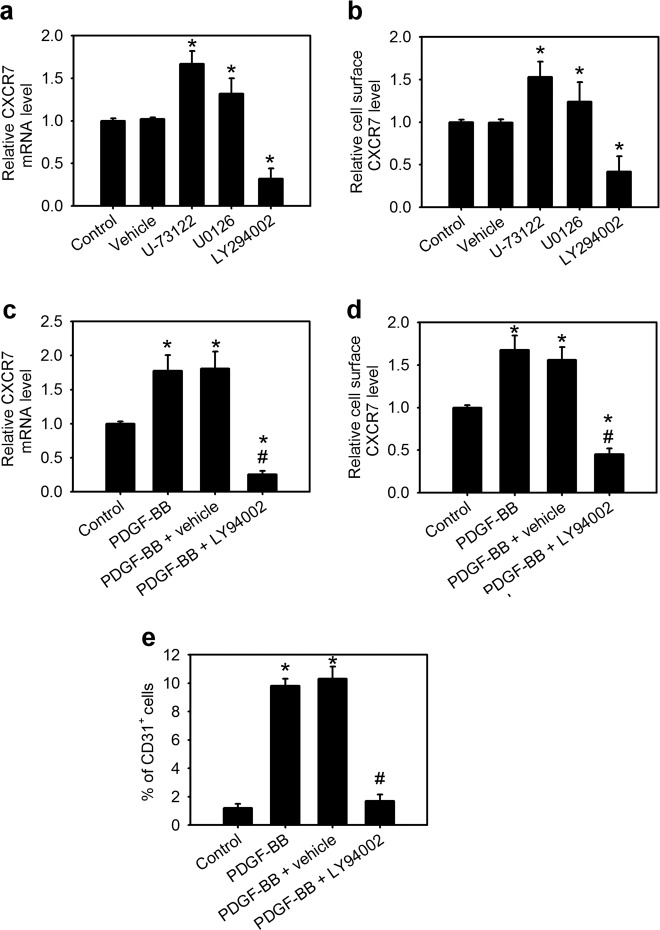
PDGFR-mediated PI3K signaling is required for VEGF or PDGF-induced CXCR7 expression. The CXCR7 mRNA levels (**a**) and its cell surface levels (**b**) in ihMSCs pretreated with vehicle (DMSO), U-73122 (phospholipase C inhibitor, 10 μM), U0126 (MEK inhibitor, 20 μM), or LY294002 (PI3K inhibitor, 10 μM) for 30 min followed by stimulation with VEGF (12 ng/ml) for 24 h. Data are means ± SD (*n* = 9). *p < 0.01 compared with the control (untreated) group. The CXCR7 mRNA levels (**c**) and its cell surface levels (**d**) in ihMSCs pretreated for 30 min with vehicle (DMSO) or LY294002 (10 μM) followed by stimulation with PDGF-BB (10 ng/ml) for 24 h. **e** The perc**e**ntage of CD31^+^ endothelial cells for ihMSCs treated with or without LY294002 followed by stimulation with PDGF-BB for 7 days. Data are means ± SD (*n* = 9). *p < 0.01 compared with the control (untreated) group. ^#^*p* < 0.0001 compared with the vehicle group.

Phospholipase C (PLC), MEK/mitogen-activated protein kinase (MEK/MAPK) and phosphatidylinositol 3-kinase (PI3K) pathways are the critical signaling cascades for PDGFRs^30^. Indeed, ihMSCs with VEGF or PDGF treatment could activate PLC, MEK/MAPK, and PI3K pathways (Supplementary Fig. S3a). We next investigated the mechanism by which PDGFRs signaling regulates the expression of CXCR7 for VEGF stimulation. ihMSCs were pretreated with specific PLC, MEK/MAPK, or PI3K inhibitors before VEGF-induced CXCR7 expression. The inhibition of the PLC with an aminosteroid phospholipase C inhibitor, U-73122, or the MEK/MAPK pathway with a selective MEK-1/2 inhibitor, U0126, caused an increase in CXCR7 expression, suggesting that PLC and MEK/MAPK pathways negatively regulate CXCR7 expression (Fig. 3a, b). However, the inhibition of the PI3K pathway with a selective PI3K inhibitor, LY294002, suppressed CXCR7 expression, indicating that PI3K acts as an activator of CXCR7 expression. Moreover, we also confirm whether PDGF, as well as VEGF, can induce CXCR7 expression. Treatment of ihMSCs with PDGF-BB significantly increased CXCR7 expression (Fig. 3c, d). However, pretreatment with LY294002 significantly decreased PDGF-BB-mediated CXCR7 expression. Moreover, the similar results were also observed in primary bone marrow-derived human MSCs (phMSCs) (Supplementary Fig. S3b, c). Altogether, we conclude that PDGFRs-mediated PI3K signaling is required for VEGF or PDGF-induced CXCR7 expression in MSCs.

### Blockage of CXCR7 inhibits VEGF-mediated vasculogenesis by MSCs

**Fig. 4:**
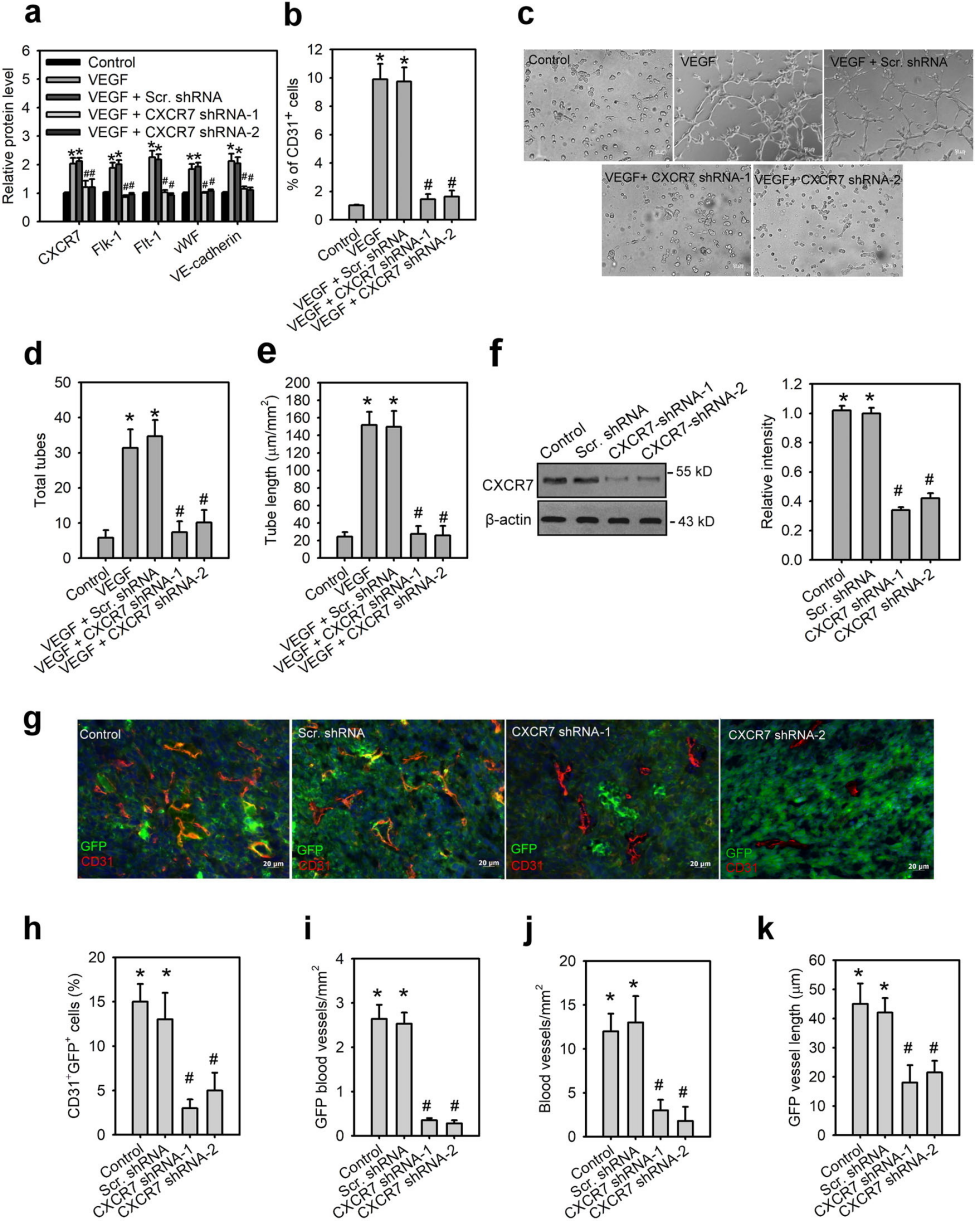
Blockage of CXCR7 inhibits VEGF-mediated vasculogenesis by MSCs. Cell surface levels of CXCR7 and endothelial cell-specific makers (**a**) and percentage of CD31^+^ endothelial cells (**b**) for ihMSCs lentivirally transduced with or without scramble (scr.) or CXCR7 shRNAs for 24 h followed by stimulation with VEGF for 7 days. Data are means ± SD (*n* = 9). *p < 0.001 compared with control medium. ^#^*p* < 0.0001 compared with scr. shRNA. The morphology characteristics (**c**) and quantification of total tubes (**d**) and tube length (**e**) for in vitro tube formation of differentiating cells derived from ihMSCs lentivirally transduced with scr. or CXCR7 shRNAs for 24 h followed by stimulation with VEGF for 7 days. Data are means ± SD (*n* = 9). *p < 0.0001 compared with control medium. ^#^*p* < 0.0001 compared with scr. shRNA. **f** Verification of CXCR7 knockdown in mouse GFP^+^MSCs. **g** Immunostaining of matrigel plugs for GFP^+^MSCs (green), CD31 (red) and DAPI (blue). Yellow, double-stained area. Quantification of CD31^+^GFP^+^ cells (**h**), GFP blood vessels (**i**), blood vessels (**j**) and GFP vessel length (**k**) in matrigel plugs. Data are means ± SD (*n* = 6). *p < 0.0001 compared with control GFP^+^MSCs without lentiviral transduction (control).

It has been reported that VEGF can induce MSCs differentiated into endothelial cells (ECs)^7^. To decipher the contribution of CXCR7 in VEGF-induced endothelial differentiation of human MSCs, we selectively silenced CXCR7 using lentiviral-based system. Flow cytometric analysis confirmed successful knockdown as manifested by significantly decreased VEGF-induced CXCR7 expression in ihMSCs (Fig. 4a). To exclude the possibility that endogenous endothelial progenitor cells (CD34^+^) and endothelial cells (CD31^+^) may contaminate the stem cells, cell sorting was carried out to remove CD34^+^ and CD31^+^ cells before VEGF treatment. ihMSCs with VEGF treatment increased the expression of endothelial-specific markers, such as fetal liver kinase receptor 1 (Flk-1), VEGF receptor 1 (Flt-1), von Willebrand factor (vWF), and vascular endothelial-cadherin (VE-cadherin) (Fig. 4a), and the frequency of generated ECs (CD31^+^; Fig. 4b). However, CXCR7 shRNA-expressing ihMSCs inhibited VEGF-induced endothelial-specific markers and CD31^+^ cells. We also tested the impact of blocking CXCR7 on VEGF-induced the capillary tube-like formation in vitro. VEGF treatment induced the ability of ihMSCs to form tube-like structures (Fig. 4c). ihMSCs with CXCR7 knockdown formed less tubes than scramble shRNA-expressing ihMSCs or wild-type ihMSCs (control ihMSCs), and the CXCR7 knockdown tubes were shorter than tubes from control ihMSCs (Fig. 4d, e). Besides, treatment of ihMSCs with a CXCR7 antagonist, CCX771, also inhibited VEGF-induced CD31^+^ cells and tube formation (Supplementary Fig. S4a–e), suggesting CXCR7 is critical mediator for VEGF-induced MSC vasculogenesis.

To confirm in vitro data, we examined vasculogenesis in vivo by performing a Matrigel plug assay. We first knocked down CXCR7 in mouse GFP^+^MSCs using CXCR7 target shRNA via a lentiviral-based system (Fig. 4f). GFP^+^MSCs with or without CXCR7 knockdown were injected s.c. into C57BL/6 mice. Two weeks later, Matrigel plugs were harvested and assayed for determination of hemoglobin concentration, endothelial differentiation, and capillary formation (Supplementary Fig. S4f). CXCR7 shRNA-expressing GFP^+^MSCs had impaired vascularization compared with scramble shRNA-expressing GFP^+^MSCs and wild-type GFP^+^MSCs (Supplementary Fig. S4g, h; Fig. 4g). Furthermore, GFP^+^MSCs with CXCR7 knockdown exhibited diminished endothelial differentiation as assessed by the percentage of vWF^+^GFP ^+^ endothelial cells in GFP + MSCs or CD31 staining colocalized with GFP, and also reduced the number of GFP^+^ blood vessels and total vessels compared with other groups (Supplementary Fig. S4h, i; Fig. 4h–j). The length of GFP^+^ blood vessels derived from GFP^+^MSCs with CXCR7 knockdown was shorter than GFP^+^ blood vessels derived from other groups (Fig. 4k). These results agree with in vitro data and support the positive effect of CXCR7 on VEGF-vasculogenesis by MSCs.

### SDF-1/CXCR7 axis promotes vasculogenesis by MSCs

**Fig. 5:**
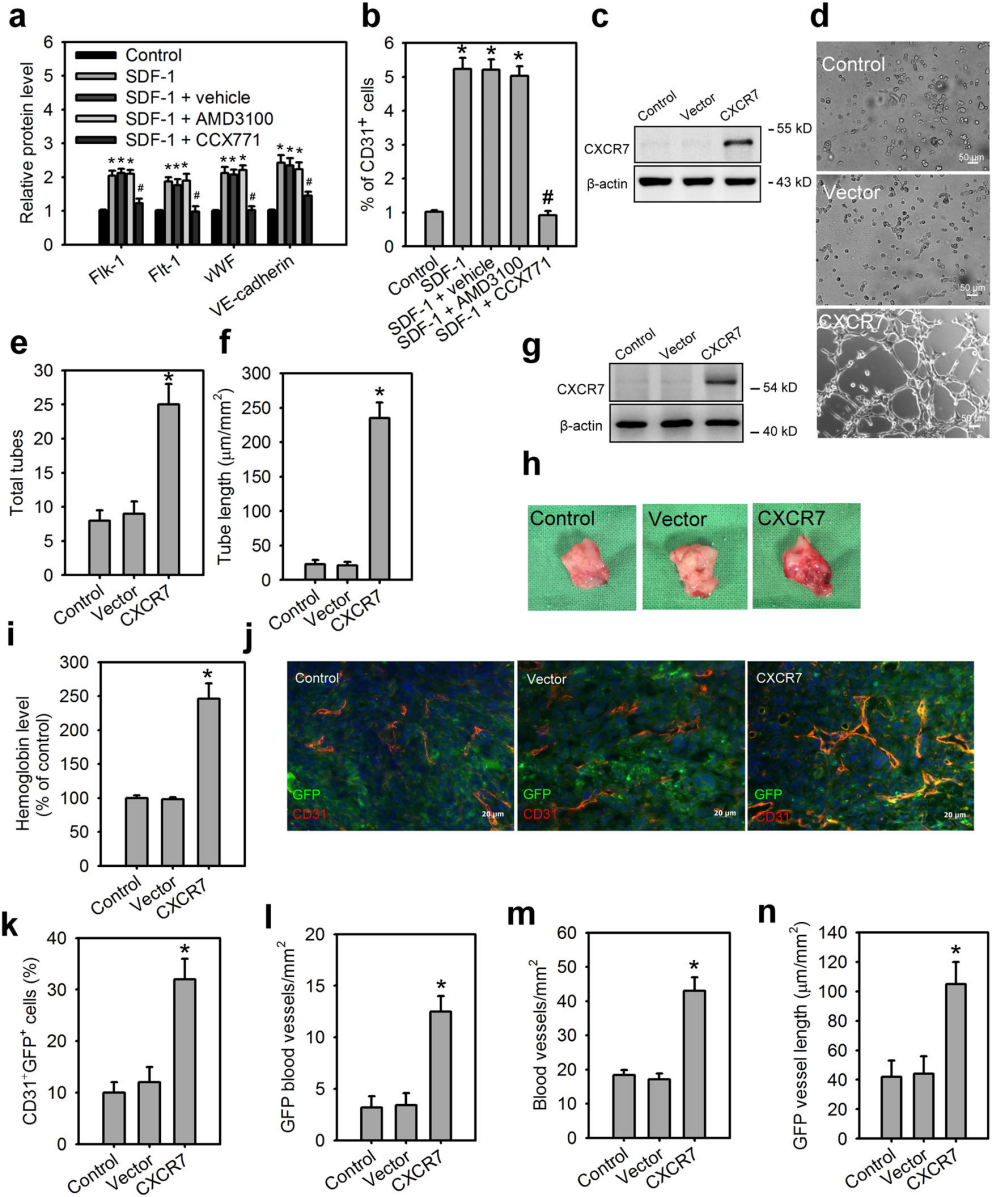
The SDF-1/CXCR7 axis promotes vasculogenesis by MSCs. Cell surface levels of endothelial cell-specific makers (**a**) and percentage of CD31^+^ endothelial cells (**b**) for ihMSCs cultured in control medium, SDF-1 medium, SDF-1 medium with vehicle DMSO, SDF-1 medium with AMD3100, and SDF-1 medium with CCX771 for 7 days. Data are means ± SD (*n* = 9). *p < 0.001 compared with control medium. ^#^*p* < 0.001 compared with SDF-1 medium with vehicle. **c** Verification of CXCR7 overexpression in ihMSCs. The morphology characteristics (**d**) and quantification of total tubes (**e**) and tube length (**f**) for in vitro tube formation of differentiating cells derived from ihMSCs lentivirally transduced with control vector or CXCR7-expressing vector for 7 days. Data are means ± SD (*n* = 9). *p < 0.0001 compared with control MSCs without lentiviral transduction (control). **g** Verification of CXCR7 overexpression in mouse GFP^+^MSCs. **h** Matrigel plugs containing wild-type GFP^+^MSCs (control), GFP^+^MSCs expressing control vector (vector) or GFP^+^MSCs expressing CXCR7 (CXCR7) subcutaneously implanted for 14 days in C57BL/6 mice. **i** Hemoglobin content in matrigel plugs. Data are means ± SD (*n* = 6). *p < 0.001 compared with control MSCs without lentiviral transduction (control). **j** Immunostaining of matrigel plugs for GFP^+^MSCs (green), CD31 (red) and DAPI (blue). Yellow, double-stained area. Quantification of CD31^+^GFP^+^ cells (**k**), GFP blood vessels (**l**), blood vessels (**m**), and GFP vessel length (**n**) in matrigel plugs. Data are means ± SD (*n* = 6). *p < 0.0001 compared with control MSCs without lentiviral transduction (control).

We next determined the role of SDF-1/CXCR4/CXCR7 in human MSC differentiation into endothelial cells (ECs). In the SDF-1-induced ihMSCs differentiation, CCX71 treatment, but not AMD3100, a specific inhibitor of CXCR4, decreased the expression of endothelial-specific markers and the frequency of generated ECs (CD31^+^; Fig. 5a, b), indicating CXCR7, but not CXCR4, plays a critical role in SDF-1-induced endothelial differentiation of MSCs. Because the SDF-1/CXCR7 axis promotes endothelial differentiation of MSCs, we test whether CXCR7 gain-of-function is able to promote vasculogenesis by MSCs. ihMSCs were stably transduced using recombinant lentiviruses expressing CXCR7 (Fig. 5c). CXCR7 gain-of-function in ihMSCs largely increased the frequency of generated ECs (CD31^+^ cells) even blockage of either cell surface PDGFRα or PDGFRβ using the neutralization antibodies (Supplementary Fig. S5). Compared with wild-type ihMSCs or control lentiviral vector-infected ihMSCs, CXCR7-expressing ihMSCs also exhibited an increase in the capillary tube-like formation in vitro (Fig. 5d–f). Moreover, GFP^+^MSCs with or without CXCR7 overexpression were injected s.c. into C57BL/6 mice for in vivo Matrigel plug assay (Fig. 5g, h). Compared with implanted wild-type GFP^+^MSCs or control vector-expressing GFP^+^MSCs groups, the implantation of CXCR7-expressing GFP^+^MSCs promoted vascularization, endothelial differentiation of GFP^+^MSCs, and vessel elongation (Fig. 5i–n), indicating CXCR7 gain-of-function promotes vasculogenesis by MSCs and its therapeutic impact on ischemic diseases.

### The SDF-1/CXCR7 axis induces the arterial specification by MSCs

**Fig. 6:**
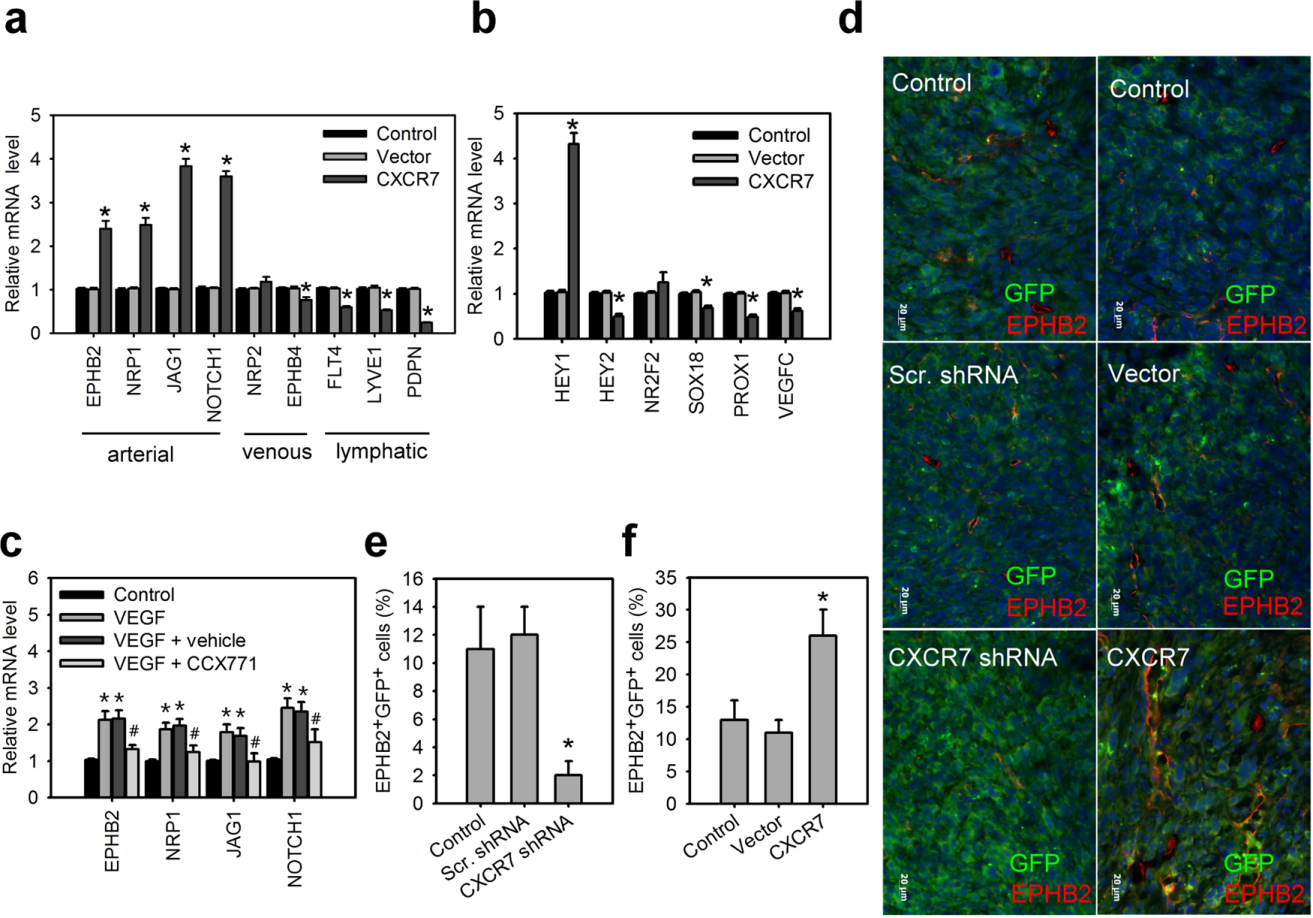
SDF-1/CXCR7 axis induces the arterial specification by MSCs. **a** The transcript levels of arterial, venous, and lymphatic markers for ihMSCs lentivirally transduced with control vector or CXCR7-expressing vector for 7 days. Data are means ± SD (*n* = 6). *p < 0.01 compared with control MSCs without lentiviral transduction (control). **b** The transcript levels of known factors regulated arterial, venous, or lymphatic endothelial cell fate for ihMSCs lentivirally transduced with control vector or CXCR7 expressing vector for 7 days. Data are means ± SD (*n* = 6). *p < 0.01 compared with control MSCs without lentiviral transduction (control). **c** The transcript levels of arterial markers for ihMSCs cultured in control medium, VEGF medium, VEGF medium with vehicle DMSO, VEGF medium with CCX771 for 7 days. Data are means ± SD (*n* = 6). *p < 0.001 compared with control medium. ^#^*p* < 0.01 compared with VEGF medium with vehicle. **d** Immunostaining of matrigel plugs for mouse GFP^+^MSCs (green), EPHB2 (red) and DAPI (blue). Matrigel plugs containing wild-type GFP^+^MSCs, GFP^+^MSCs expressing control vector, GFP^+^MSCs expressing CXCR7, GFP^+^MSCs expressing scramble (scr.) shRNA, or GFP^+^MSCs expressing CXCR7 shRNA were subcutaneously implanted for 14 days in immunocompromised mice (NOD-SCID). Yellow, double-stained area. **e**, **f** Quantification of EPHB2^+^GFP^+^ cells in matrigel plugs. Data are means ± SD (*n* = 6). *p < 0.001 compared with GFP^+^MSCs without lentiviral transduction (control).

Because endothelial differentiation can be along the arterial, venous, and lymphatic EC lineages^31,32^, we further determined which lineage is involved in the SDF-1/CXCR7 axis-mediated endothelial differentiation. Q-PCR analysis revealed that CXCR7 gain-of-function in ihMSCs promoted the expression of cell surface markers in arterial ECs, such as ephrin type-B receptor 2 (EPHB2), neuropilin 1 (NRP1), jagged1 (JAG1), and notch homolog 1(NOTCH1), compared with control lentiviral vector-infected ihMSCs or wild-type ihMSCs (Fig. 6a). However, CXCR7 gain-of-function resulted in downregulation or no significant difference in venous or lymphatic EC markers, including neuropilin 2 (NRP2), ephrin type-B receptor 4 (EPH4), vascular endothelial growth factor receptor 3 (FLT4), lymphatic vessel endothelial receptor 1 (LYVE1), and podoplanin (PDPN). CXCR7 gain-of-function in ihMSCs also largely induced hairy/enhancer-of-split related with YRPW motif protein 1 (HEY1) expression (Fig. 6b), which is required for arterial cell fate and vascular development^33–35^. However, no significant difference of COUP transcription factor 2 (NR2F2) expression, which is a transcriptional mediator of venous EC cell specification^36^, and downregulation of transcription factor SOX-18 (Sox18), prospero homeobox protein 1 (Prox1), and vascular endothelial growth factor C (VEGFC), which are required for specifying lymphatic endothelial cell fate, were observed in ihMSCs with CXCR7 gain-of-function. Moreover, VEGF stimulation also increased the arterial EC markers in ihMSCs and the frequency of arterial ECs (EPHB2^+^ cells) in phMSC (Fig. 6c; Supplementary Fig. S4b). However, blocking CXCR7 by CCX71 significantly inhibited VEGF-induced the expression of the arterial EC markers and the frequency of arterial ECs. The immunofluorescence imaging also demonstrated that mouse GFP^+^MSCs with CXCR7 knockdown showed diminished arterial EC differentiation as assessed by arterial EC marker (EPHB2) staining colocalized with GFP in vivo Matrigel plug assay. However, the implantation of CXCR7-expressing GFP^+^MSCs were colocized with EPHB2 (Fig. 6d–f; Supplementary Fig. S6). These finding clearly suggest that the SDF-1/CXCR7 axis promotes the arterial specification by MSCs.

### Activation of Notch signaling is a critical mechanism for CXCR7-mediated the arterial specification by MSCs

**Fig. 7:**
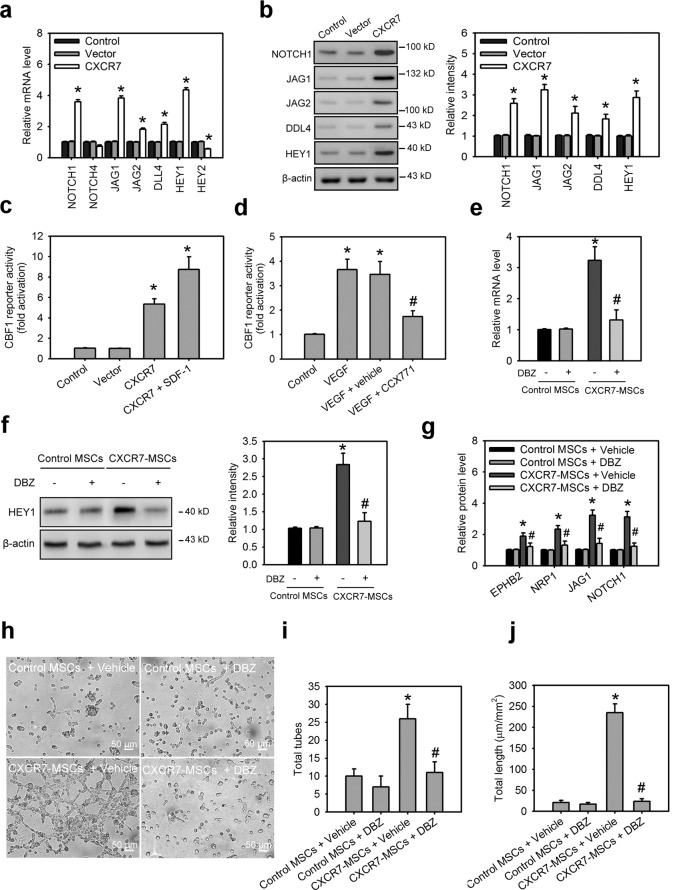
Activation of Notch signaling is a critical mechanism for CXCR7-mediated the arterial specification by MSCs. **a** The transcript levels of Notch receptors, their cognate ligands, and the downstream transcription factors for ihMSCs with or without CXCR7 overexpression. **b** The protein levels of Notch 1, JAG1, JAG2, DLL4, and HEY1 for ihMSCs with or without CXCR7 overexpression. Data are means ± SD (*n* = 6). *p < 0.01 compared with control MSCs without lentiviral transduction (control). **c** The CBF1 reporter activities for ihMSCs with or without the SDF-1/CXCR7 axis gain-of-function for 48 h. Data are means ± SD (*n* = 9). *p < 0.01 compared with control MSCs without lentiviral transduction (control). **d** The CBF1 reporter activities for cultured in control medium, VEGF medium, VEGF medium with vehicle DMSO, and VEGF medium with CCX771 for 48 h. Data are means ± SD (*n* = 9). *p < 0.0001 compared with control medium. ^#^*p* < 0.001 compared with VEGF medium with vehicle. **e**, **f** The transcript and protein levels of HEY1 for CXCR7-expressing MSCs and control vector-expressing MSCs (control MSCs) with or without DBZ (10 μM) treatment for 24 h. Data are means ± SD (*n* = 6). *p < 0.0001 compared with control MSCs. ^#^*p* < 0.001 compared with vehicle treatment. **g** The protein levels of arterial EC markers for CXCR7-expressing MSCs and control vector-expressing MSCs (control MSCs) with or without DBZ treatment for 7 days. Data are means ± SD (*n* = 6). *p < 0.001 compared with control MSCs. ^#^*p* < 0.01 compared with vehicle treatment. The morphology characteristics (**h**) and quantification of total tubes (**i**) and tube length (**j**) for in vitro tube formation of differentiating cells derived from control MSCs or CXCR7-expressing MSCs followed by treatment with DBZ for 7 days. Data are means ± SD (*n* = 9). *p < 0.0001 compared with control MSCs. ^#^*p* < 0.0001 compared with vehicle treatment.

During vasculogenesis, Notch signaling plays an important role in the development of arterial endothelial cells^32,37^. the Notch receptors 1 and 4, their cognate ligands Jagged1 (JAG1), Jagged2 (JAG2), and delta-like ligand 4 (DLL4), and the downstream transcription factors, HEY1 and HEY2, contribute to the Notch signaling-mediated arterial specification. Therefore, we determined and compared these gene expressions in CXCR7-expressing ihMSCs, control lentiviral vector-infected ihMSCs, and wild-type ihMSCs by Q-PCR. Interestingly, Notch 1 receptor, JAG1, JAG2, DLL4, and HEY1 were upregulation in CXCR7-expressing ihMSCs, but not in control lentiviral vector-infected ihMSCs or wild-type ihMSCs (Fig. 7a). Western blot analysis also confirmed their protein levels, and had the similar results (Fig. 7b). Besides, the Human Notch Signaling Pathway Plus RT² Profiler PCR Array comprised an 84 gene panel involved in Notch signaling was applied to ihMSCs with or without CXCR7 overexpression. Among these genes, over twofold change of upregulation was only found in Notch 1 receptor, JAG1, and DLL4, suggesting these molecules are the dominant mediators to induce Notch signaling under CXCR7 gain-of-function (Supplementary Fig. S7). We next further investigated whether the SDF-1/CXCR7 axis induces the activation of Notch signaling. To examine Notch activity in MSCs, we transfected luciferase reporter constructs with or without four Notch-sensing CBF1-binding sites upstream of an SV40 promoter into ihMSCs. In the absence of co-expressing CXCR7, the CBF1 reporter had no activity above the basal level of the SV40 promoter (Fig. 7c); however, in the presence of CXCR7 overexpression, the CBF1 reporter exhibited a ~eightfold increase in activity. Furthermore, VEGF treatment also increased the reporter activity in ihMSCs. However, blocking CXCR7 by CCX71 significantly suppressed the VEGF-induced the reporter activity in MSCs (Fig. 7d). To elucidate the Notch signaling plays a role in CXCR7-mediated HEY1 expression and arterial specification by ihMSCs. The γ-secretase inhibitor, dibenzazepine (DBZ), was used to inhibit Notch signaling. Treatment of CXCR7-expressing ihMSCs but not control vector-expressing ihMSCs with DBZ significantly inhibited the expression of HEY1 expression (Fig. 7e, f) and arterial EC markers (EPHB2, NRP1, JAG1, and NOTCH1) (Fig. 7g). DBZ treatment also blocked CXCR7-promoted capillary tube-like formation in ihMSCs (Fig. 7h–j), and VEGF induced the frequency of ECs (CD31^+^ cells) or arterial ECs (EPHB2^+^ cells) and tube formation in phMSC (Supplementary Fig. S4a–e). Taken together, these findings indicate that the SDF-1/CXCR7 axis can induce activation of Notch signaling via upregulation of Notch 1 receptor and its cognate ligand, JAG1. CXCR7-activated Notch signaling further increases HEY1 expression, which contributes to vasculogenesis and the arterial specification by MSCs.

## Discussion

Knockdown of CXCR7 in zebrafish showed a defect in vessel formation during development, suggesting CXCR7 plays critical roles in developmental vasculogenesis and angiogenesis^17^. Owing to CXCR7 levels in normal stem cells and endothelial cells are very low, its roles in postnatal and pathological vasculogenesis are poorly understood^15^. Here, we provide a novel function of CXCR7 in postnatal vasculogenesis and arterial specification. CXCR7 promotes endothelial differentiation of MSCs and postnatal vasculogenesis. The most specifically, CXCR7 controls the arterial EC differentiation and arterial formation through activation of Notch signaling. The CXCR7-mediated Notch activation triggers an upregulation of a key transitional factor, HEY1, and this mechanism may play a central role in CXCR7-induced arterial EC differentiation and arterial development because HEY1 required for arterial cell fate and vascular development^33–35^. Moreover, gain of CXCR7 function does not change the expression of the transcription factor COUP-TFII, which is a transcriptional mediator of venous EC cell specification^36^, but inhibits the induction of SOX-18, PROX1, and VEGFC, which are required for specifying lymphatic endothelial cell fate^38,39^, suggesting CXCR7 controls cell fate in arterial endothelial cells but, not in venous and lymphatic endothelial cells. Altogether, these data represent the first evidence that CXCR7 acts as a novel mediator in endothelial differentiation and arterial specification via modulation of Notch signaling.

In order to elucidate the role of CXCR7 in postnatal and pathological vasculogenesis, we first suspected that VEGF is an important inducer for CXCR7 expression in stem cells during vasculogenesis. VEGF activates CXCR7 expression in tumor cells and endothelial cells through supposed VEGF receptors and their downstream signaling pathways^40,41^. However, this notion is not suitable for MSCs because these cells have no VEGFR expression, suggesting other receptors are requirement for CXCR7 induction^11^. The previous study identified an alternative receptor, PDFGR, for VEGF-A-regulated MSC migration and proliferation, indicating the PDGFR signaling is able to replace the VEGFR signaling for VEGF interaction in cells lacking VEGFR^11^. Our results reveal the same mechanism is required for VEGF-mediated CXCR7 expression in MSCs, and both PDGFRα and PDGFRβ are dominant functional receptors for this event. We also found that the downstream signaling pathway of PDGFRs, PI3K, is critical for CXCR7 regulation. Importantly, we identified another angiogenic factor, PDGF-BB, also has the same function in regulation of CXCR7, suggesting PDGF/PDGFR singling mechanism plays a role in CXCR7 induction. Therefore, further studies seem to be needed to investigate its impact on the regulation of endothelial or tumor CXCR7.

Hypoxia can upregulate CXCR7 expression in MSCs in vitro^40^. Blockade of hypoxia-inducible factor (HIF)-1α inhibited hypoxia-promoted CXCR7, suggesting HIF-1α can modulate CXCR7 expression. However, it is still unclear that hypoxic activation of CXCR7 expression is through direct or indirect action of HIF-1α, since the functional data on CXCR7 promoter regulation by HIF-1α were not available so far. Here, we found that CXCR7 expression was significantly elevated in MSCs cultured in CM from hypoxia-treated SkMC. In addition, the neutralizing antibodies of VEGF inhibited hypoxic CM or tissue ischemia-induced CXCR7 expression on MSCs in vitro or transplanted MSCs in vivo, suggesting VEGF is a critical mediator for hypoxic microenvironment-activated CXCR7 expression. Owing to VEGF is a HIF-1α target gene^42^, HIF-1α may indirectly regulate CXCR7 expression by VEGF involved in the mechanism of hypoxia-mediated upregulation of CXCR7.

Several studies have uncovered the functional role of endothelial CXCR7 in survival, proliferation, migration, and tube formation of mature ECs or its progenitor cells^40,43–45^. However, its roles on endothelial differentiation and specification are unclear. By this study, we show that CXCR7 is not only required for VEGF-induced endothelial differentiation by MSCs but also is sufficient for triggering endothelial differentiation of MSCs when they acquire the gain of function from genetic engineering. Interestingly, CXCR7 further largely promotes arterial EC differentiation, but has no role in venous and lymphatic EC differentiation. Manipulation of CXC7 in MSCs triggers a change in arterial markers, but has no effect on venous and lymphatic markers. Moreover, implanted MSCs with CXCR7 genetic engineering were coloziated with cells expressing arterial marker in vivo. These findings provide a specific evidence that CXCR7 controls the differentiation of adult stem cells into arterial endothelial cells, and this mechanism further contributes to postnatal vasculogenesis. It would be interesting to investigate whether CXCR7 also has a role in controlling endothelial differentiation and specification, thereby regulating plasticity of vascular specification. Further studies are needed to clarify these mechanisms in embryonic stem cells.

This work provides a novel singling mechanism that CXCR7 activates Notch singling. This mechanism arises from gain of CXCR7 function in CXCR7 low-expressing MSCs upregulates the expression of Notch receptors 1 and 4, their cognate ligands JAG1, JAG2, and DLL4, and further triggers Notch activation. Subsequently, blockage of CXCR7 also partially inhibits VEGF-induced Notch singling on MSCs, suggesting CXCR7 plays a role on VEGF-induced Notch signaling. Although the detail molecular mechanism of CXCR7-mediated Notch signaling remains uncertain, our results show a cross talk between CXCR7 and Notch signaling pathways. CXCR7 activation is able to induce Notch signaling via upregulation of Notch receptors and their ligands. However, it is unclear whether the SDF-1/CXCR4 axis, as well as SDF-1/CXCR7 axis, is able to activate the Notch signaling. Besides, the previous study demonstrated that Notch signaling controls the expression of SDF-1 and its receptor, CXCR4, and functions in myeloma cell lines and MSCs^46^. It is also interestingly test whether Notch singling can regulate the expression and function of CXCR7.

The capability to obtain and expand ECs via ex vivo differentiation of MSCs makes these cells promising candidates for vascular regenerative therapies. The main obstacle is the challenge of precisely controlling differentiation into ECs while producing sufficient quantities. In this study, we show that the SDF-1/CXCR7 axis has a profound influence on endothelial differentiation of MSCs, and that it can maximize efficiency of EC production through this process. A previous study has shown that MSCs can differentiate into ECs in the presence of SDF-1 and that MSCs overexpressing SDF-1 can produce effective angiogenesis^47^. In this study, treatment with additional exogenous SDF-1 promoted MSC differentiation into ECs, but the frequency of generated ECs was low (~4%). Surprisingly, we found that CXCR7 overexpression in MSCs greatly enhanced ECs production by ~eightfold during exogenous SDF-1-induced MSC differentiation. Therefore, CXCR7 gain-of-function in transplanted MSCs may be an attractive therapeutic approach that can significantly alter ischemic disease outcome via vasculogenesis.

In summary, these data provide conclusive evidence that the cross talk between stromal cells and MSCs via VEGF or PDGF-mediated PI3K/Akt signaling induces CXCR7 expression on MSCs. Notch signaling is a critical downstream pathway for CXCR7-mediated postnatal vasculogenesis and arterial specification. These mechanisms may further contribute to tumor growth or tissue repair. These findings also have important implications for therapeutic application of postnatal vasculogenesis to ischemic disorders.

## Supplementary information

Supplementary Tables

Supplementary Figure Legends

Supplementary Figure S1

Supplementary Figure S2

Supplementary Figure S3

Supplementary Figure S4

Supplementary Figure S5

Supplementary Figure S6

Supplementary Figure S7
