# Supplementary material for: Atypical chemokine receptor ACKR3/CXCR7 controls postnatal vasculogenesis and arterial specification by mesenchymal stem cells via Notch signaling
Source: Cell Death Dis. 2020 May 4;11(5):307. doi: 10.1038/s41419-020-2512-2 (PMC7198625; doi:10.1038/s41419-020-2512-2)
Supplement: Supplementary file 1 — Supplementary Tables [file 41419_2020_2512_MOESM1_ESM.docx]

**Supplementary Tables**

**Atypical chemokine receptor ACKR3/CXCR7 controls postnatal vasculogenesis and arterial specification by mesenchymal stem cells via Notch signaling**

Sung-Tai Wei^1,2^, Yen‐Chih Huang^1,4^​, Mei-Ling Hsieh^3^, Yu-Jung Lin^1^, Woei-Cherng Shyu^3^, Hui-Chen Chen^1,3^, Chia-Hung Hsieh^1,5,6^

^1^Graduate Institute of Biomedical Sciences, China Medical University, Taichung, Taiwan

^2^Department of Neurosurgery, China Medical University and Hospital, Taichung, Taiwan

^1^Graduate Institute of Immunology, China Medical University, Taichung, Taiwan

^4^Department of Medical Imaging, China Medical University and Hospital, Taichung, Taiwan

^5^Department of Medical Research, China Medical University Hospital, Taichung, Taiwan

^6^Department of Biomedical Informatics, Asia University, Taichung, Taiwan

**Table S1.** primer sets for q-PCR

**Table S2.** shRNA target sequences

| Gene | Target sequence |
| --- | --- |
| Human CXCR7  Mouse CXCR7 | GCCAGGGAACTTCTCGGACAT for shRNA-1  GACACGGTGATGTGTCCCA for shRNA-2  CTTCACTATCATTGCGATCTT for shRNA-1  GCCTGGCAACTACTCTGACAT for shRNA-2 |
