# Supplementary material for: Atypical chemokine receptor ACKR3/CXCR7 controls postnatal vasculogenesis and arterial specification by mesenchymal stem cells via Notch signaling
Source: Cell Death Dis. 2020 May 4;11(5):307. doi: 10.1038/s41419-020-2512-2 (PMC7198625; doi:10.1038/s41419-020-2512-2)
Supplement: Supplementary file 2 — Supplementary Figure Legends [file 41419_2020_2512_MOESM2_ESM.docx]

**Supplementary Figure Legends**

**Atypical chemokine receptor ACKR3/CXCR7 controls postnatal vasculogenesis and arterial specification by mesenchymal stem cells via Notch signaling**

Sung-Tai Wei^1,2^, Yen‐Chih Huang^1,4^​, Mei-Ling Hsieh^3^, Yu-Jung Lin^1^, Woei-Cherng Shyu^3^, Hui-Chen Chen^1,3^, Chia-Hung Hsieh^1,5,6^

^1^Graduate Institute of Biomedical Sciences, China Medical University, Taichung, Taiwan

^2^Department of Neurosurgery, China Medical University and Hospital, Taichung, Taiwan

^1^Graduate Institute of Immunology, China Medical University, Taichung, Taiwan

^4^Department of Medical Imaging, China Medical University and Hospital, Taichung, Taiwan

^5^Department of Medical Research, China Medical University Hospital, Taichung, Taiwan

^6^Department of Biomedical Informatics, Asia University, Taichung, Taiwan

**Supplementary Figure S1.** The VEGF levels in ischemic limbs and plasma of mice with or without limb ischemia. Tissues and plasma were analyzed the VEGF levels by ELISA at 2 days after limb ischemia. Data are means ± SD (n=6). *p < 0.01 compared to Non-treated group.

**Supplementary Figure S2.** (**a**) Protein levels and relative protein densities of VEGFR in ihMSCs and HUVEC. Data are means ± SD (n=9). *p < 0.0001 compared to the ihMSCs. (**b**) The CXCR7 protein levels and relative protein densities in ihMSCs pretreated with the neutralizing anti-PDGFRα antibody, anti-PDGFRβ antibody or both followed by stimulation with VEGF (12 ng/ml) for 24 h. Data are means ± SD (n=9). *p < 0.001 compared to the control (untreated) group. ^#^p < 0.01 compared to IgG-treated groups. (**c**) The relative protein densities of PDGFRα and PDGFRβ expression in ihMSCs with or without PDGFRα or PDGFRβ knockdown by siRNAs. Data are means ± SD (n=6). *P < 0.001 compared with scramble (Scr.) siRNA.

**Supplementary Figure S3.** (**a**) Immunoblotting for phospho- or total PLC-γ1, MEK-1/2 and Akt in ihMSCs treated with PDGF or VEGF for 24 h. Data are means ± SD (n=9). *p < 0.0001 compared to the control (untreated) group. The CXCR7 mRNA levels (**b**) and its cell surface levels (**c**) in phMSCs pretreated with or without vehicle (DMSO) or LY294002 (PI3K inhibitor, 10 μM) for 30 min followed by stimulation with VEGF (12 ng/ml) or PDGF-BB (10 ng/ml) for 24 h. (**d**) The percentage of CD31^+^ endothelial cells for phMSCs treated with or without LY294002 followed by stimulation with PDGF-BB for 7 days. Data are means ± SD (n=9). *p < 0.0001 compared to the control (untreated) group. ^#^p < 0.0001 compared to vehicle treated group.

**Supplementary Figure S4.** The percentage of CD31^+^ endothelial cells (**a**) and EPHB2^+^ endothelial cells (**b**) for phMSCs treated with or without vehicle (DMSO), CCX771 (100 nM) or DBZ DBZ (10 μM) followed by stimulation with VEGF for 7 days. Data are means ± SD (n=9). *p < 0.0001 compared to the control (untreated) group. ^#^p < 0.0001 compared to vehicle treated group. The morphology characteristics (**c**) and quantification of total tubes (**d**) and tube length (**e**) for *in vitro* tube formation of differentiating cells derived from phMSCs treated with or without vehicle (DMSO), CCX771 (100 nM) or DBZ DBZ (10 μM) followed by stimulation with VEGF for 7 days. Data are means ± SD (n=9). Data are means ± SD (n=9). *p < 0.0001 compared to the control (untreated) group. ^#^p < 0.0001 compared to vehicle treated group. (**f**) Matrigel plugs containing wild-type GFP^+^MSCs, GFP^+^MSCs expressing scr. shRNA or GFP^+^MSCs expressing CXCR7 shRNA subcutaneously implanted for 14 days in C57BL/6 mice. (**g**) Hemoglobin content in matrigel plugs. Data are means ± SD (n=6). *P < 0.001 compared with control GFP^+^MSCs without lentiviral transduction (control). (**h**) The percentage of vWF^+^GFP ^+^ endothelial cells in GFP^+^MSCs with or without CXCR7 knockdown isolated from matrigel plugs via a flow sorting of GFP-expressing cells. Data are means ± SD (n=6). *P < 0.001 compared with control GFP^+^MSCs without lentiviral transduction (control). (**i**) Immunostaining of matrigel plugs for GFP^+^MSCs (green) and CD31 (red).

**Supplementary Figure S5.** The percentage of CD31^+^ endothelial cells for ihMSCs lentivirally transduced with or without CXCR7 and cultured in control medium, SDF-1 medium, SDF-1 medium with IgG, SDF-1 medium with the neutralizing anti-PDGFRα antibody or SDF-1 medium with the neutralizing anti-PDGFRβ antibody followed by stimulation with VEGF (12 ng/ml) for 7 days. Data are means ± SD (n=9). *p < 0.001 compared to the control (untreated) group.

**Supplementary Figure S6.** Immunostaining of matrigel plugs for mouse GFP^+^MSCs (green) and EPHB2 (red). Matrigel plugs containing wild-type GFP^+^MSCs, GFP^+^MSCs expressing control vector, GFP^+^MSCs expressing CXCR7, GFP^+^MSCs expressing scramble (scr.) shRNA or GFP^+^MSCs expressing CXCR7 shRNA were subcutaneously implanted for 14 days in immunocompromised mice (NOD-SCID).

**Supplementary Figure S7.** The Human Notch Signaling Pathway Plus RT² Profiler PCR Array comprised of an 84 gene panel involved in Notch signaling was applied to ihMSCs with or without CXCR7 overexpression. Data are means ± SD (n=3).
