# Supplementary figures and images for: Atypical chemokine receptor ACKR3/CXCR7 controls postnatal vasculogenesis and arterial specification by mesenchymal stem cells via Notch signaling
Source: Cell Death Dis. 2020 May 4;11(5):307. doi: 10.1038/s41419-020-2512-2 (PMC7198625; doi:10.1038/s41419-020-2512-2)

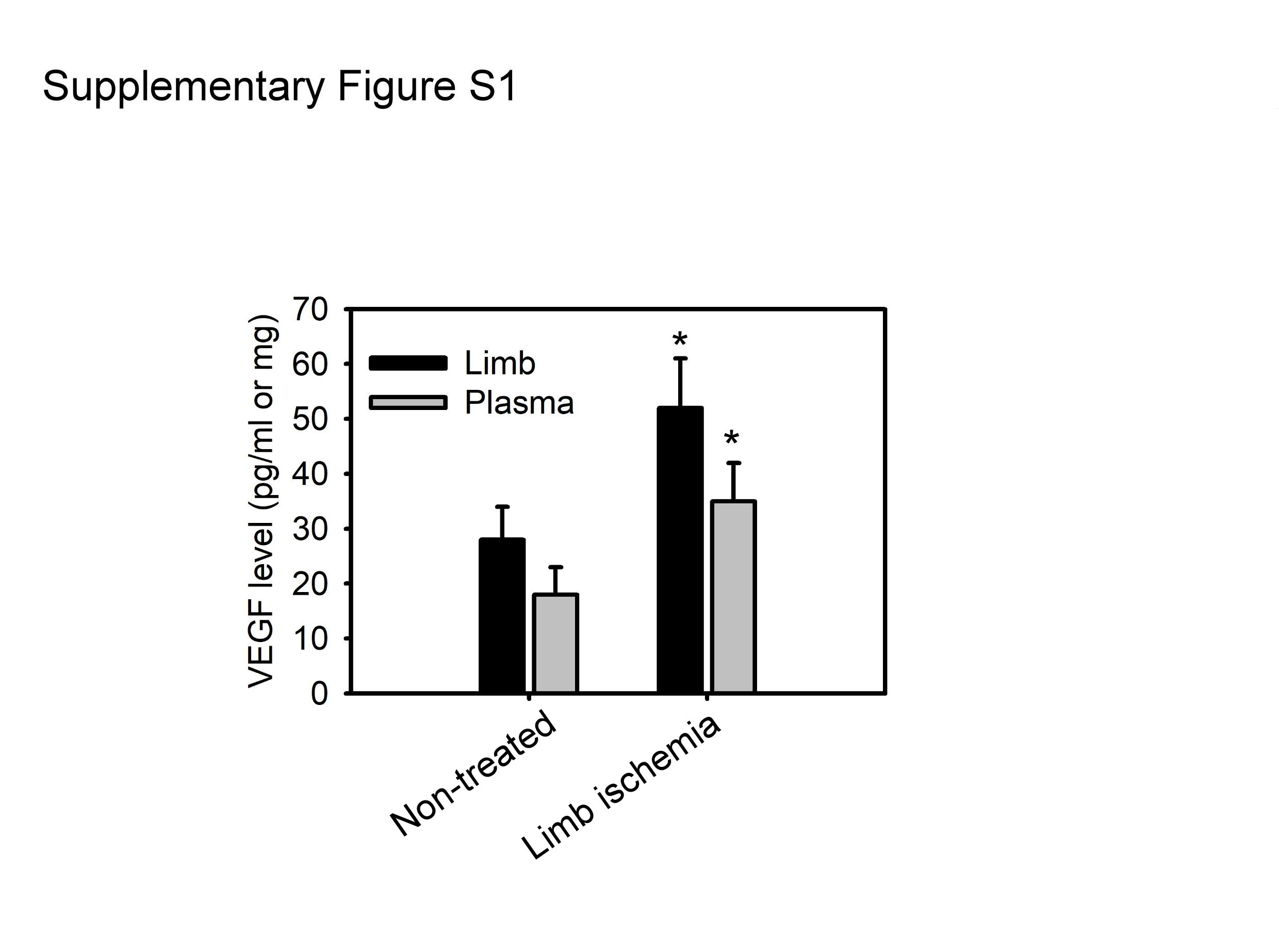

Supplement: Supplementary file 3 — Supplementary Figure S1 [file 41419_2020_2512_MOESM3_ESM.png]

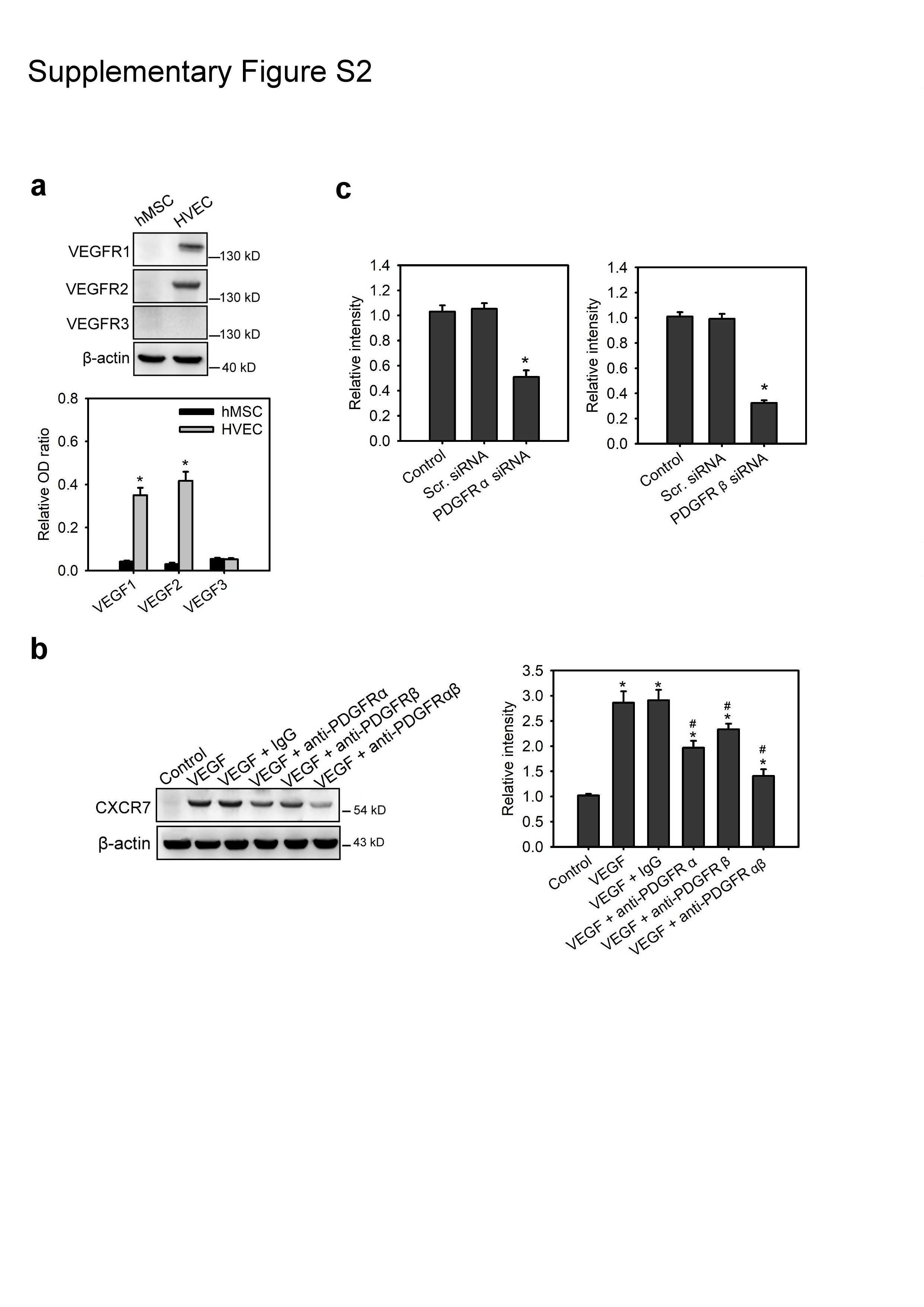

Supplement: Supplementary file 4 — Supplementary Figure S2 [file 41419_2020_2512_MOESM4_ESM.png]

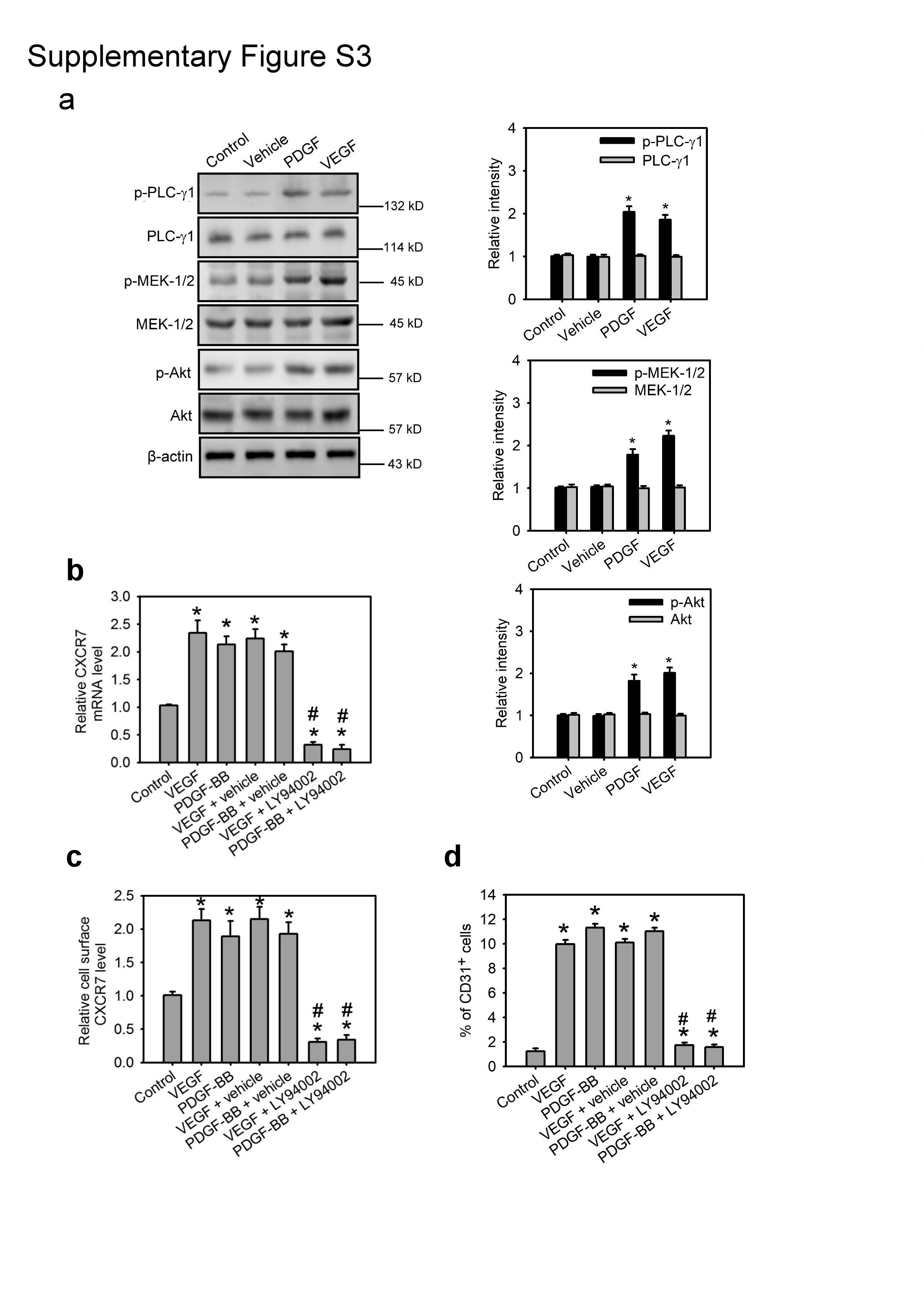

Supplement: Supplementary file 5 — Supplementary Figure S3 [file 41419_2020_2512_MOESM5_ESM.png]

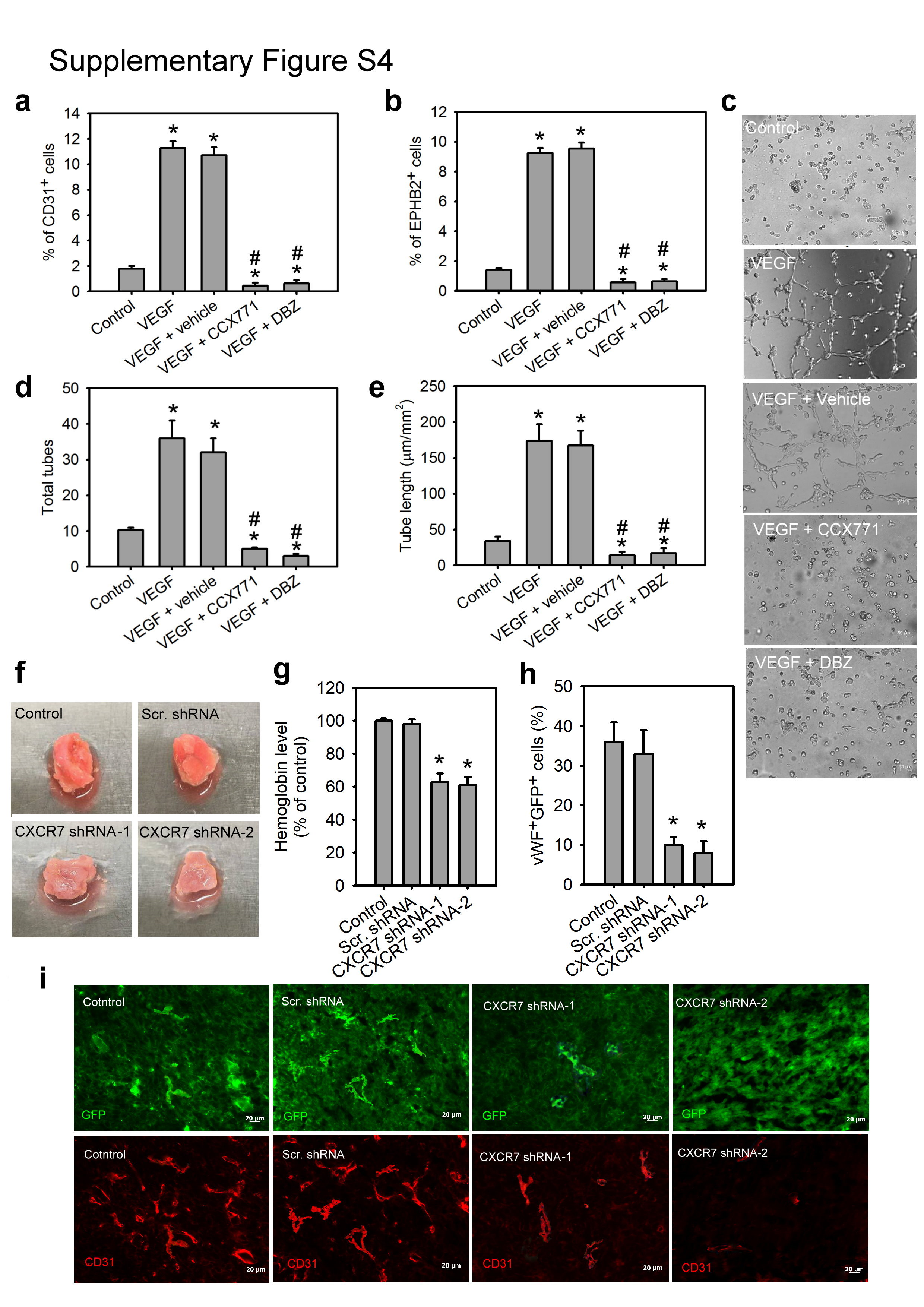

Supplement: Supplementary file 6 — Supplementary Figure S4 [file 41419_2020_2512_MOESM6_ESM.png]

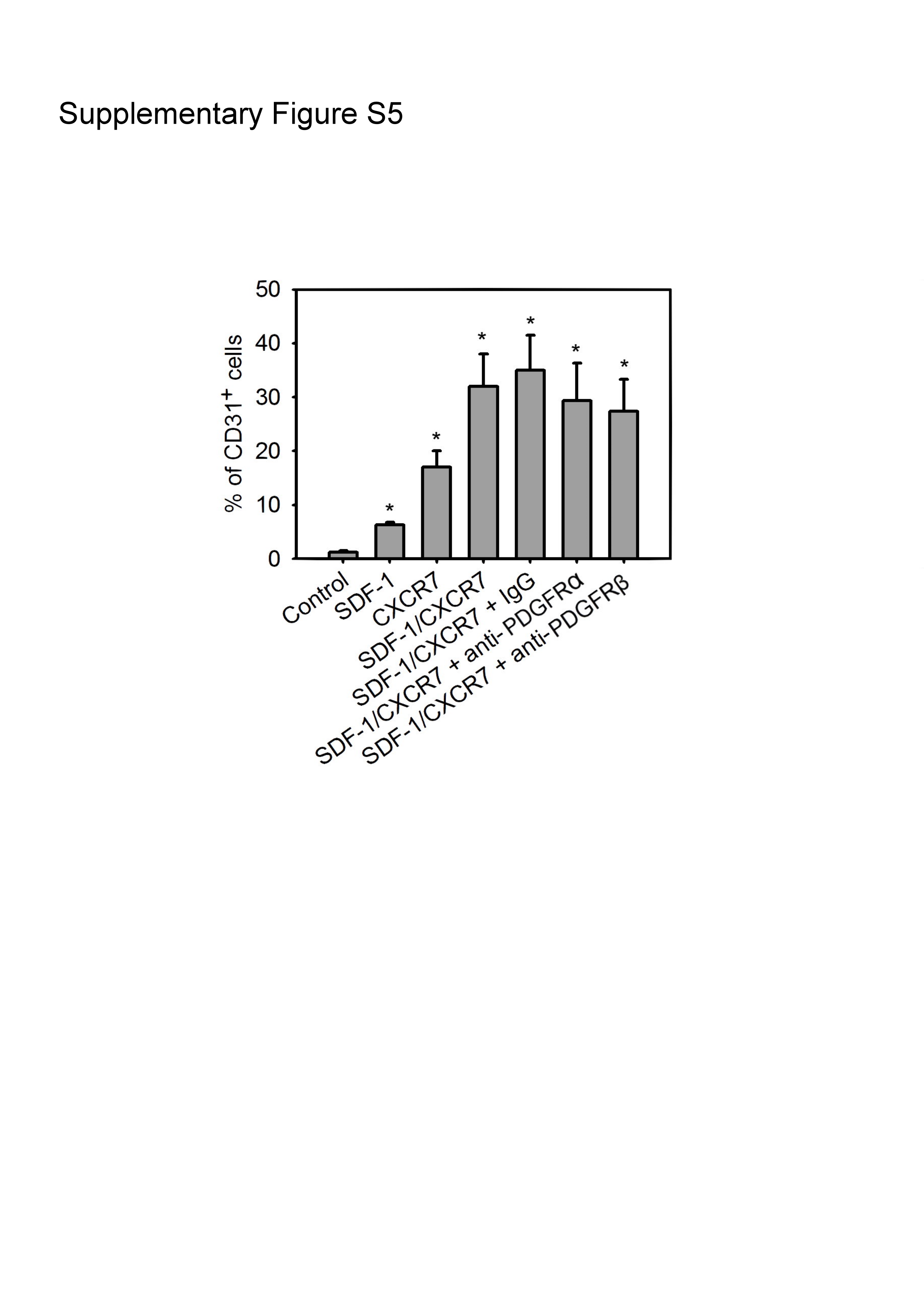

Supplement: Supplementary file 7 — Supplementary Figure S5 [file 41419_2020_2512_MOESM7_ESM.png]

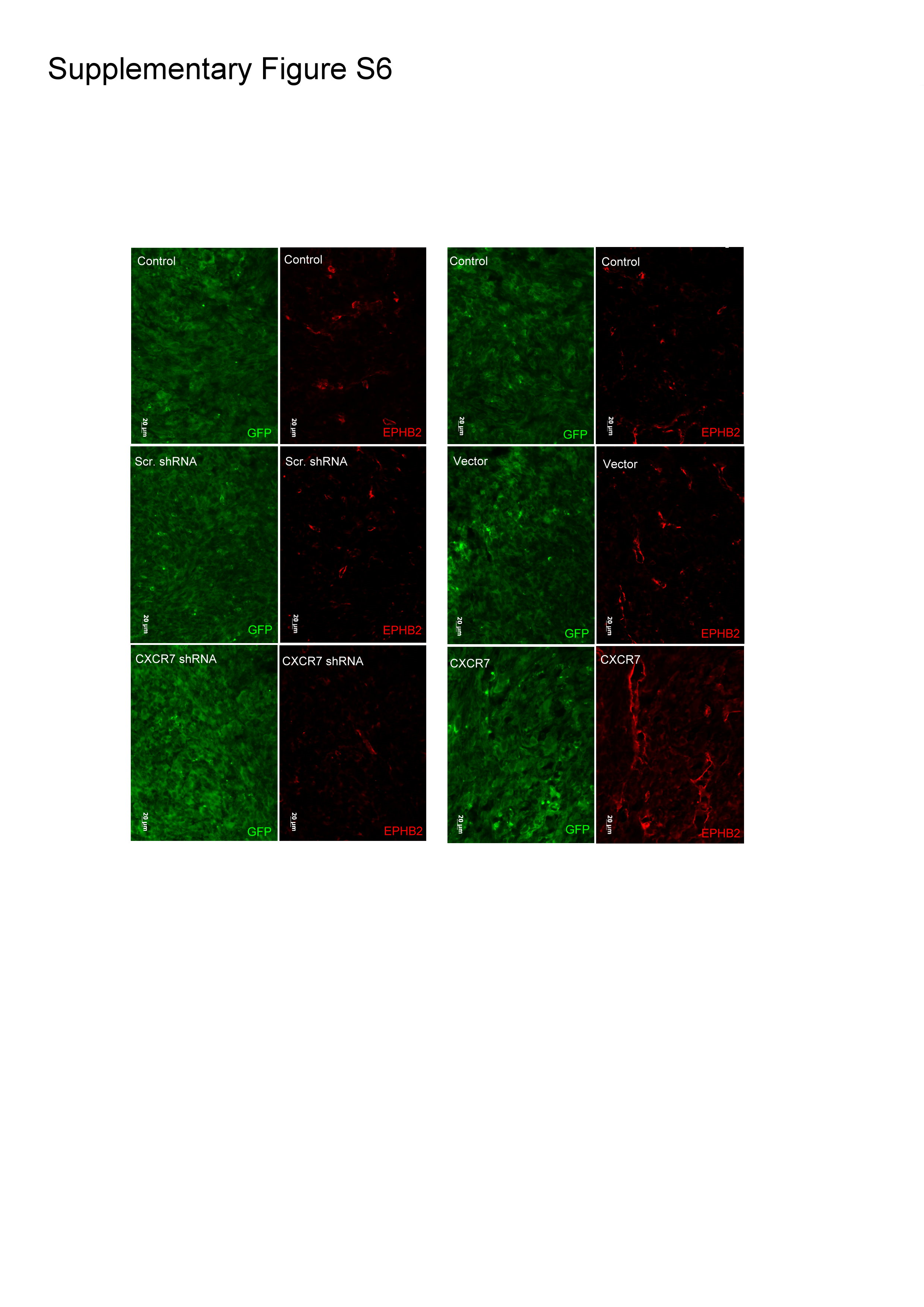

Supplement: Supplementary file 8 — Supplementary Figure S6 [file 41419_2020_2512_MOESM8_ESM.png]

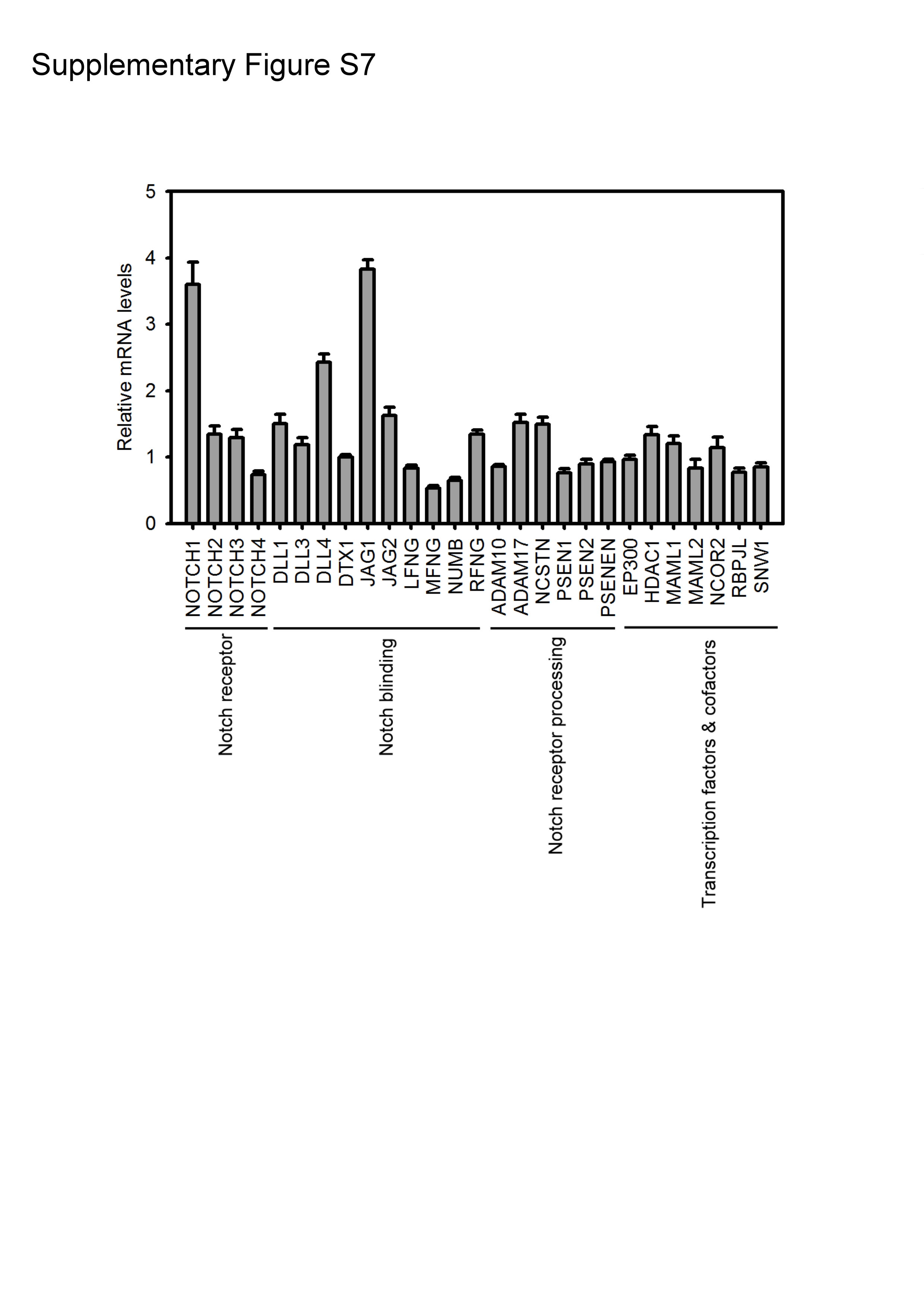

Supplement: Supplementary file 9 — Supplementary Figure S7 [file 41419_2020_2512_MOESM9_ESM.png]
